# Comparative Analysis of 2022 Outbreak MPXV and Previous Clade II MPXV

**DOI:** 10.1002/jmv.70023

**Published:** 2024-10-28

**Authors:** Joseph Patrick McGrail, Alberto Paniz Mondolfi, Juan David Ramírez, Santiago Vidal, Adolfo García‐Sastre, Gustavo Palacios, Mari Paz Sanchez‐Seco, Susana Guerra

**Affiliations:** ^1^ Department of Preventive Medicine, Public Health and Microbiology Universidad Autónoma de Madrid Madrid Spain; ^2^ Department of Pathology, Molecular, and Cell‐Based Medicine Icahn School of Medicine at Mount Sinai New York New York USA; ^3^ Centro de Investigaciones en Microbiología y Biotecnología‐UR (CIMBIUR), Facultad de Ciencias Naturales Universidad del Rosario Bogotá Colombia; ^4^ Department of Microbiology Icahn School of Medicine at Mount Sinai New York USA; ^5^ Global Health and Emerging Pathogens Institute Icahn School of Medicine at Mount Sinai New York USA; ^6^ Department of Medicine, Division of Infectious Diseases Icahn School of Medicine at Mount Sinai New York New York USA; ^7^ The Tisch Cancer Institute Icahn School of Medicine at Mount Sinai New York New York USA; ^8^ Department of Pathology, Molecular and Cell‐Based Medicine Icahn School of Medicine at Mount Sinai New York USA; ^9^ The Icahn Genomics Institute Icahn School of Medicine at Mount Sinai New York New York USA; ^10^ Centro Nacional de Microbiología Instituto de Salud Carlos III Madrid Spain; ^11^ Centro de Investigación Biomédica en Red de Enfermedades Infecciosas (CIBERINFEC) Instituto de Salud Carlos III Madrid Spain

**Keywords:** Clade II, EV, MPOX disease, MPXV, MV

## Abstract

The 2022–2024 outbreak of MPOX is an important worldwide public health issue that has triggered significant concerns in the scientific community. MPOX is caused by monkeypox virus (MPXV) belonging to the *Poxviridae* family. The study of MPXV presents a multifaceted challenge due to the diverse viral formThis study was supported by ISIDORe consortium and Agencia Estatal de Investigación.s produced by this pathogen. Notably the intracellular mature viruses (MVs) primarily contribute to localized lesions and host‐to‐host transmission, while the extracellular enveloped viruses (EVs) are associated with systemic infection. Clinically, MPOX manifests as a vesiculopustular rash that initially emerges on the face and trunk, subsequently spreading throughout the body, with heightened severity observed in immunocompromised individuals. Results obtained in this manuscript indicate that the 2022 outbreak MPXV has a significantly slower viral cycle compared with previous Clade II strains, with WRAIR 7‐61 being more intermediate and USA 2003 producing highest viral titers. Additionally, proteomic and phospho‐proteomic analysis displays differences in protein expression between these three strains. These findings highlight key differences between the current Lineage B.1 MPXV and previous strains. Further studies will be undertaken to demonstrate if these differences are important for the apparent increased human‐to‐human transmission mechanisms observed in the Clade IIb MPXV outbreak.

## 1 Introduction

Monkeypox virus (MPXV) is a member of the *Poxviridae* Family (genus *Orthopoxvirus*) and the cause of the infectious disease known as Monkeypox (MPOX) [1, 2]. Poxviruses contain a double‐stranded DNA genome that is approximately 190 kilobase‐pairs in length. This genome contains over 200 genes that encode numerous proteins which permits the virus to have all the necessary machinery to replicate in the cytoplasm instead of the cell nucleus, contrary to most other DNA viruses [3, 4]. Another *Orthopoxvirus* characteristic is their intricate morphogenetic pathway, which leads to the production of three different viral forms, intracellular mature virus (MV), cell‐associated enveloped virus (CEV), and extracellular enveloped virus (or EV) during the viral cycle [5]. MVs mediate host‐to‐host transmission through the formation of localized lesions, while EVs are associated with systemic infection within the host [6, 7]. CEVs induce the production of actin tails to assist viral infection of other cells [8].

The origins of MPXV can be traced back to 1958 where the virus was identified as causing a pox‐like disease in captive monkeys transported from Africa [2, 9]. In 1970, a 9‐month‐old boy from Zaire (now known as the Democratic Republic of Congo) was the first documented case of MPOX in humans [10]. Multiple outbreaks of MPOX were reported afterwards but were limited to Central and Western Africa [11]. Despite their name, rodents appear to be the main reservoir of MPXV. It was not until 2003 that MPXV came heavily under the spotlight when 73 cases were reported primarily in Wisconsin, United States by the CDC [12]. These cases were thought to have originated through close contact with prairie dogs that were infected by rodent species imported from Africa [9]. The potential danger that MPXV could cause worldwide was now more evident due to this being the first time that human MPXV infections were reported outside of Africa.

During this timeframe, MPXV evolved into two distinct clades: Clade I and Clade II. Clade I MPX originates from Central Africa and causes more pronounced mortality, morbidity, and viraemia, with a case‐fatality ratio (CFR) > 10%. Meanwhile, Clade II MPXV is associated with Western Africa and is much less virulent than Clade I MPXV, usually associated with a CFR < 1% [1, 13, 14]. Clade II MPXV eventually derived into Clade IIa and Clade IIb. The USA 2003 outbreak MPXV and the endemic infections in Western Africa belong to Clade IIa, while the global 2022 outbreak MPXV belongs to Lineage B.1 of Clade IIb [15, 16].

The Clade IIb MPOX outbreak is an important public health issue that has led to 97 745 cases and 203 fatalities being reported across 116 countries up to May 2024 [17]. Lineage B.1 MPXV is characterized primarily by a much higher reproduction number in humans (*R*) than previous strains, with a value of *R* > 1 [1, 18]. This value is even greater when taking only into account risk groups such as men who have sex with men (MSM), with *R* values of approximately 2 [19]. Clinical features include a vesiculopustular rash that spreads from the face and trunk to the rest of the body after 12–16 days from exposure. The rash morphologically develops progressively into macular, papular, vesicular, and pustular lesions [1, 20, 21]. Immunocompromised patients develop much more severe features, including necrotizing skin lesions, secondary infections, and sepsis. This is especially relevant in regard to the MSM population with advanced HIV infection that is more at risk of severe MPOX infection [22]. In addition, cases of infection in vaccinated individuals are being reported recently, suggesting even further caution in regard to MPXV [23].

In this study, using mouse embryonic fibroblast (MEF) cells, a comparative analysis of Clade II MPXV was undertaken to better understand what attributes Lineage B.1 MPXV, that caused the 2022 MPOX outbreak, possesses that are different from strains associated with previous outbreaks. WRAIR 7–61 and USA 2003 were selected due to representing two distinct timepoints in Clade II MPXV evolution, with the first being a 1961 Western Africa MPXV isolate from an infected macaque and the second belonging to the 2003 Wisconsin human outbreak, with both belonging to Clade IIa. Meanwhile, the recent strain was isolated from an infected patient of the 2022 MPOX Lineage B.1 outbreak. Results obtained demonstrate a slower viral cycle of Lineage B.1 MPXV compared with previous Clade II strains and significantly different protein expression in infected cells. A higher extracellular infectious virus titer of the 2003 USA outbreak MPXV compared with the 2022 MPXV strain could indicate that a lessened EV production could be associated with the higher dissemination rate in MPXV Lineage B.1. In addition, proteomic analysis of infected cells with the three MPXV strains displays distinct patterns of protein expression, which might also have a role in explaining the higher magnitude and transmissibility of the Clade IIb MPXV outbreak.

## 2 Materials and Methods

### 2.1 Cells and Viruses

Immortalized MEFs (kindly provided by K.P Knobeloch) were cultured in 1 g/L glucose Dulbecco Modified Eagle Medium (DMEM) supplemented with penicillin (100 U/mL; Sigma‐Aldrich, St. Louis, MO, USA), streptomycin (100 μg/mL; Sigma‐Aldrich), l‐glutamine (2 mM; Sigma‐Aldrich), nonessential amino acids (0.1 mM; Sigma‐Aldrich), and 5% heat‐inactivated fetal bovine serum (FBS) (Invitrogen Gibco). BSC40 (ATCC CRL‐2761) cells were cultured in the same conditions except they were supplemented with 10% FBS. Cell cultures were maintained at 37°C in a humidified incubator containing 5% CO_2_. MPXV strain corresponding to a human isolate from the 2022 MOPX outbreak used for this work had been isolated from fluid (vesicle) content from a clinical skin (lesion) swab specimen (PV67610) [24] and its titer previously obtained in BSC‐40 cells (data not shown). USA 2003 and WRAIR 7‐61 viruses were obtained through BEI Resources.

### 2.2 Viral Stock Generation

All virus work was performed in a BSL3 facility by trained scientists wearing appropriate personnel protective equipment. Fourteen confluent BSC‐40 F‐175 were inoculated at MOI 0.01 with each MPXV strain. When the cytopathic effect was evident, the infected cells were scraped in the flask medium with a cell scraper, and the resulting suspension was transferred to sterile 20 mL centrifugation tubes. Infected cells were pelleted by centrifugation at 2000*g* and 4°C for 5 min. Supernatant was discarded, and each pellet was resuspended in 1 mL of DMEM and added to the same centrifugation tube. After centrifugation, the supernatant was discarded once again, and the pellet was resuspended in 2–3 mL DMEM. Suspension was aliquoted, freeze–thaw cycle was conducted three times and titration was done by standard plaque assay.

### 2.3 Virus Titration

Extracellular titer was measured through obtaining the supernatant of infected cells after the designated hpi (24 and 48 hpi), while intracellular titer was measured through scraping the infected cells in 0.5 mL of DMEM. In addition, intracellular virus samples were subjected to three cycles of freezing–thawing to allow for the cell lysis. Intracellular and extracellular virus was quantified using a standard plaque assay [25]. BSC‐40 cells were seeded to obtain confluence in an M12 plate on the day of titration. 1/10 dilution series of virus samples are prepared and inoculated into the cells of different wells with standard infection protocol. Cells are left in liquid infection medium and fixated with PFA 4% and stained with Crystal Violet‐PFA after 48 hpi. Lysis plaques are counted and viral titer is obtained through plaque assay formula.

### 2.4 Protein Analysis by Western Blot Analysis

Infected Inmortalized MEFs lysates were lysed through the addition of 100 µL of SDS‐Laemmli sample buffer supplemented with 100 mM dithiothreitol (DTT) to each M12 well plate. Cell lysates were boiled for 5 min, resolved by sodium dodecyl sulfate‐polyacrylamide gel electrophoresis (SDS‐PAGE) with Laemmli running buffer and transferred to polyvinylidene difluoride membranes (Merck‐Millipore) in a Trans‐Blot SD Semi‐Dry Transfer Cell (Bio‐Rad) according to the manufacturer's recommendations. Membranes were blocked with 5% skim milk in PBS containing 0.1% Tween 20 (PBS‐T) and incubated with the corresponding primary antibodies as indicated in the figure legends Beta‐actin was obtained from Santa Cruz. Regarding to the viral antibodies (E3 was generously provided by Bertram L. Jacobs, F13 by Rafael Blasco and A4 and D8 by Mariano Esteban), incubation was undertaken in 0.5% skim milk in PBS‐T. Membranes were then washed with PBS‐T and incubated with anti‐rabbit, anti‐mouse, or anti‐rat peroxidase‐labeled antibodies (1:10 000; Sigma‐Aldrich). After extensive washing with PBS‐T, the immune complexes were detected by using Clarity Western ECL blot substrate (Bio‐Rad) and a ChemiDoc XRS^+^ System (Bio‐Rad), according to the manufacturer's instructions.

### 2.5 Immunofluorescence

Immortalized MEFs were grown on 12‐mm‐diameter glass coverslips in DMEM–5% FCS to a confluence of 40%–50% and mock‐infected or infected at a multiplicity of infection (MOI) of 0.1 PFU/cell and 1 PFU/cell with the different Clade II MPXV strains. At 24 hpi, cells were washed with PBS, fixed with 4% paraformaldehyde, permeabilized with 0.25% Triton X‐100 in PBS for 30 min, and blocked in PBS with 10% FCS for 30 min at room temperature. Poli‐VACV (Invitrogen PA1‐7258), a policlonal antibody was used as the primary antibody. Alexa Fluor 488‐conjugated rabbit IgG antibodies (Invitrogen) were used as secondary antibodies. Cell nuclei were stained with 4′,6′‐diamidino‐2‐phenylindole (DAPI; Sigma, 1:200). Fluorescent microscopy was performed using a Leica DM4 B Microscope, and images were collected and processed with LAS X software (Leica, Wetzlar, Germany).

### 2.6 Urea 8 M Buffer Lysis of Infected Cells for Proteomics

Buffer was prepared by adding 12.02 g of urea and 16 mL of 50 mM NH_4_HCO_3_. F‐75 flask of MEF cells were seeded to obtain 90% confluence on the day of infection. After the standard infection procedure with each MPXV strain, the medium was removed 21 hpi and 500 µL of Urea 8 M buffer was used to lyse cells with the help of a cell scraper. Lysate is transferred to eppendorfs and maintained at 4°C.

### 2.7 Tryptic Digestion and Desalting of Samples

The total concentration of homogenates is measured with Bio‐Rad Protein Assay Dye. A total of 50 μg of total protein from each sample is carried forward for total proteomics. The urea concentration is diluted from 1 M to 100 mM NH4HCO3. Then, samples are reduced with 5 mM Tris (2‐carboxyethyl) phosphine hydrochloride (alkalinized with 10 mM idoacetamide) at room temperature, and digested with trypsin at 37°C for 16 h using Modified Trypsin Grade Sequencing. After digestion, samples are acidified with 10% trifluoroacetic acid and desalted with BioPureSPN PROTO 300 C18 Mini columns according to the manufacturer's instructions. After the samples are desalted, they are dried in a concentrating centrifuge. The dried peptides are reconstituted in 30 μL Buffer A (0.1% (vol/vol) TFA, 1% (vol/vol) acetonitriphyl in HPLC‐grade water) and analyzed on a timsTOF Pro2 mass spectrometer.

### 2.8 Mass Spectrometry Data Analysis

Desalted samples are analyzed using a hybrid trapped ion mobility quadrupole TOF mass spectrometer through a CaptiveSpray nano‐electrospray ion source (Bruker Daltonics). Hybrid trapped ion mobility quadrupole TOF mass spectrometer through a CaptiveSpray nano‐electrospray ion source (Bruker Daltonics). MS analysis will be performed in positive ion mode with the dia‐PASEF method with samples with data optimized by data independent analysis (DIA) scan parameter. We will perform DDA in PASEF mode with pooled samples of each sample type to adjust dia‐PASEF optimized parameters for each sample type. The DIA acquisition windows will be assembled separately for phospho‐enriched samples due to the known drift in mass and ion mobility of phospho‐modified peptides. To analyze diaPASEF data, raw data (.d) will be processed with DIA‐NN v18.0 [26] using spectral library generated by UniProt proteome.

### 2.9 Statistical Analysis of Proteomics Data

The input document to advance DIA data analysis is the DIA‐NN Report.pg.matrix. For data pre‐processing a proprietary R script is used. Raw intensity values are log2 transformed and normalized by median. Afterwards, missing values are inserted using QRILC imputation. For sample group comparisons, *p*‐values are calculated using the student's t‐test using the Python package scipy [27] and adjusted using the Benjamini–Hockberg method through a Statsmodels package [28]. VolcaNoseR software was used to represent volcano plots containing proteomic data [29].

### 2.10 Pathway Analysis of Proteomics Data

ShinyGOv0.80 was used to measure biological process pathway enrichment after input of MPXV‐infected MEF cellular protein true values [30]. Fold enrichment, FDR and *n* values were used to construct a plot with the use of GraphPad 9.

### 2.11 Statistical Analysis

One‐way ANOVA tests were used to analyze the differences in mean values between groups with the use of GraphPad 9. All results are expressed as means ± the standard deviations (SD). Significant differences are defined as:

*p ≤ 0.05; **p ≤ 0.005; ***p ≤ 0.001, ****p ≤ 0.0001.

## 3 Results

### 3.1 2022 MPXV Possesses Diminished Viral Growth and Different Plaque Phenotype Compared to Previous Clade II MPXV at Low Multiplicity Infection

**Figure 1:**
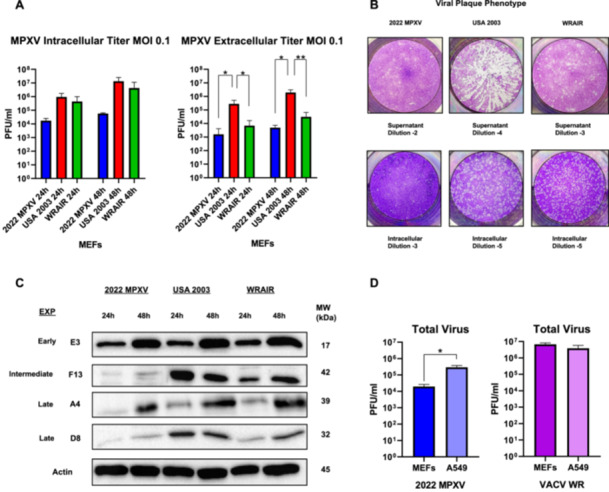
Low multiplicity infection demonstrates differences in viral growth and protein expression of 2022 MPXV compared with USA 2003 and WRAIR 7‐61. (A) MEFs were infected with the three MPXV strains (MOI 0.1), at 24 and 48 hpi, infectious viral particles from supernatants (extracellular virus) and cell extracts (intracellular virus) were titrated by plaque assay. Mean ± SD values from four independent experiments are represented. (B) Viral plaque phenotype of different clade II strains (2022 MPXV, USA 2003, and WRAIR 7‐61). Plaques were obtained through titration with BSC‐40 cells of intracellular virus and supernatant at dilutions indicated. (C) MEFs were infected with the three MPXV strain (MOI 0.1) and at the indicated times postinfection, equal amounts of proteins from cell extracts were analyzed by Western Blot. Specific antibodies for VACV early protein E3, intermediate protein F13, and late proteins D8 and A4 were used. β‐Actin was used as a loading control. Molecular weights (MW) in kilodaltons (kDa) are indicated, based on protein standards. (D) MEFs and A549 human cell lines were infected with 0.1 MOI of 2022 MPXV and VACV WR. Total virus produced at 24 hpi was quantified through plaque assay. Means ± the SD from three independent experiments are represented. *, *p* < 0.05; **, *p* < 0.01; ***, *p* < 0.005; ****, *p* < 0.0001.

The first step in our comparative analysis of 2022 MPXV and previous Clade II strains, USA 2003 and WRAIR, was to perform a viral growth curve infecting at low multiplicity (MOI 0.1). Analysis of intracellular titer indicated that 2022 MPXV displays a trend of slower viral growth in comparison to both USA 2003 and WRAIR 7–61 (Figure 1A). Meanwhile, the extracellular titer of USA 2003 is significantly different from 2022 MPXV and WRAIR at 24 and 48 hpi. Two characteristics were noteworthy when analyzing viral plaques formed. First, during titration of the intracellular virus, plaque sizes were significantly different between the three Clade II strains. 2022 MPXV produced very small plaques, meanwhile, WRAIR formed intermediate‐sized plaques and USA 2003 larger plaques. Second, during extracellular titration of USA 2003, comet‐shaped plaques were formed that were only visible in this strain (Figure 1B), confirming the previously observed increased EV production following USA 2003 infection (Figure 1A). Evaluation of viral protein quantity present in the different infections through western blot analysis validated what was observed in our viral growth experiments. In general, the higher quantity of viral proteins were present in USA 2003 and WRAIR 7‐61 infection. F13 is an intermediate protein which is in very high amount in USA 2003 at 24 hpi and slightly less at 48 hpi, while a noticeable amount is also present in WRAIR at 24 and 48 hpi, 2022 MPXV infected cells have a very small amount of F13 at both timepoints, though slightly more at 48 hpi. D8 is a late protein that is barely present at 48 hpi in 2022 MPXV, while there is a distinguishable amount in cells infected with USA 2003 and WRAIR 7‐61 at both timepoints. Although, it is worth noting that A4, a late structural core protein, is present in a reduced but more similar quantity in 2022 MPXV when compared with the other two Clade II strains (Figure 1C).

To further complement this study, a low multiplicity infection replication assay in a human cell line (A549) was conducted with the 2022 strain and a poxvirus reference strain, Vaccina Virus Western Reserve (WR) strain. Results obtained indicate that 2022 MPXV replicates better in a human cell line, with a log increase in comparison to the titer values in MEFs at 24 hpi. On the other hand, VACV WR did not present significant differences in viral growth when comparing murine and human cell lines, although it replicates at a significantly higher level, displaying that 2022 MPXV has a slower viral cycle in comparison to VACV WR (Figure 1D).

**Figure 2:**
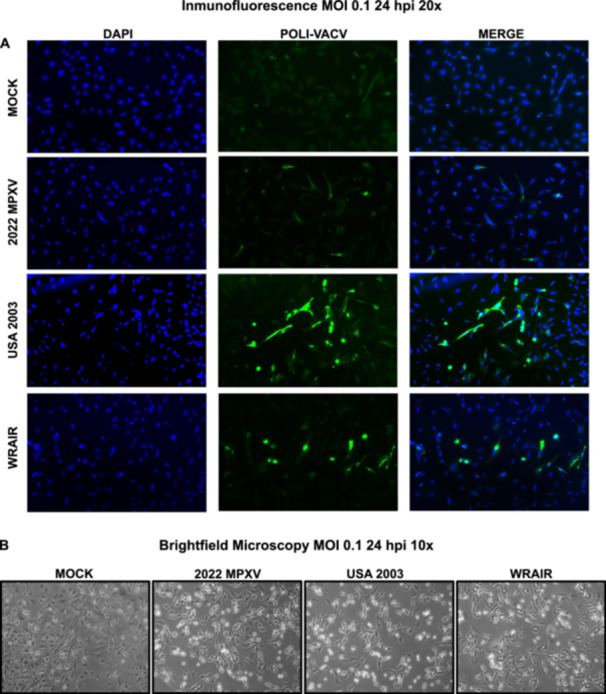
Microscopy analysis of low multiplicity MPXV infected MEF cells at 24 hpi. (A) Fluorescence microscopy analysis of MEFs infected with the three MPXV strains (MOI 0.1, 24 hpi). Infected cells were visualized using Poli‐VACV Rabbit Antibody (in green), DAPI (4′,6‐diamidino‐2‐phenylindole) (in blue) was used to stain the nuclear DNA. The experiment has been carried out in triplicate and a representative image is shown. Objective used was ×20. (B) Brightfield microscopy was used to visualize the cytopathic effect caused by each virus. The objective used was ×10. The experiment has been carried out in triplicate and a representative image is shown.

In addition, we used immunofluorescence to validate the presence and localization of viral proteins over time following infection (MOI 0.1) of MEF cells by the three strains. At 24 phi, the results of the microscopy analysis confirm a delayed infection by the new MPXV strain 2022 MPXV. Specifically, USA 2003 shows the highest number of infected cells, WRAIR slightly less and 2022 MPXV shows the least number of infected 2022 MPXV cells (Figure 2A). However, under these infection conditions, no clear difference was observed among the three virus strains in the cytopathic effect produced after infection of the immunofluorescence crystals (Figure 2B). Low multiplicity infections with the three strains did not lead to very noticeable cytopathic effects during experiments, although USA 2003 was slightly more visible. This is especially noticeable in comparison to other poxvirus reference strains, such as VACV WR, which replicates much faster and produces much more cytopathic effect.

As the infection spread in cell cultures is related to the number of infectious virus particles per cell, we decided to carry out a similar experiment using a MOI of 1 in which the majority of the cells in the culture are infected. In this condition, there are no significant differences between 2022 MPXV and the other Clade II strains, regardless of quantifying intracellular or supernatant titer (Figure S1A). USA 2003 displays a trend of slightly more intracellular titer than 2022 MPXV and WRAIR 7‐61, but there is barely any difference in supernatant titer (Figure S1A). When analyzing protein expression through western blot, the quantity of F13 and D8 is still superior in USA 2003 and WRAIR 7‐61 infection. Meanwhile, A4 and E3 are still in similar amount in 2022 MPXV infected cells compared with the other two strains even in these conditions (Figure S1B). These results suggest that the observed delay in the replication cycle of the 2022 MPXV strain can be overcome by a higher viral load. As we will discuss later, this may have a significant impact on the transmission dynamics of this new variant.

### 3.2 Proteomic Analysis Indicates Distinct Protein Expression of 2022 MPXV, USA 2003, and WRAIR 7‐61

**Figure 3:**
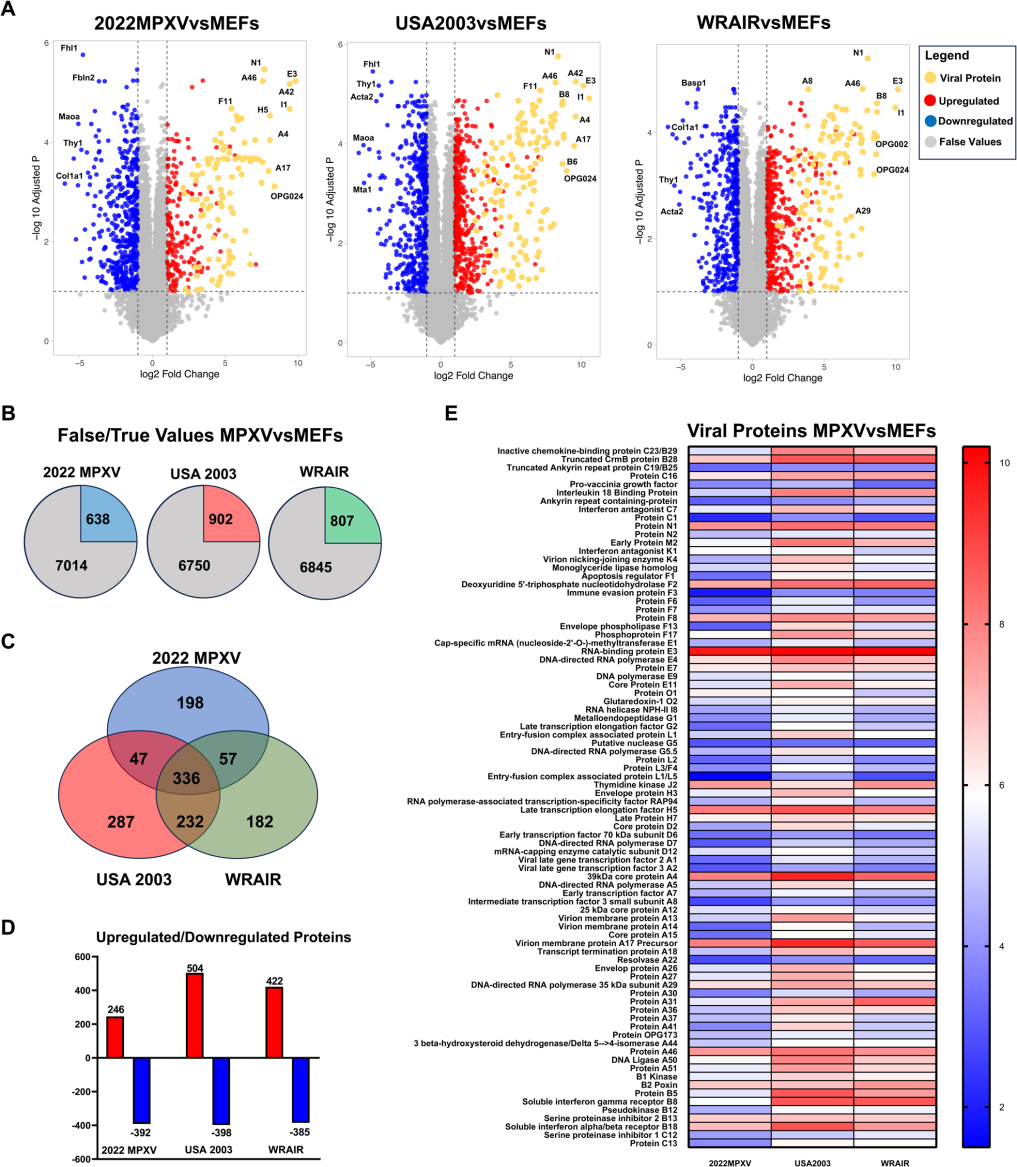
Proteomic analysis displays differential protein expression between 2022 MPXV previous Clade II strains. (A) Volcano Plot representing Log2 Fold Change and Adjusted *p* Value proteins in infected MEF cells with clade II MPXV strains compared with noninfected MEFs. Upregulated proteins are colored red, downregulated blue, viral proteins are orange, and false values are gray. Most relevant hits are labeled. (B) Pie chart displaying the number of true and false values identified. (C) Venn diagram of differentially expressed proteins across MPXV strain infection in MEF cells. (D) Graph indicating quantity of upregulated/downregulated proteins (E) Heat Map displaying Log2 Fold Change values of viral proteins in infected MEF cells with Clade II MPXV strains compared with noninfected MEFs. Experiments were conducted with four replicates of infected protein extracts.

To investigate whether the specific dissemination characteristics of the 2022 MPXV strain could be due to differences in cellular and viral protein expression compared to historical Clade II strains, we performed a quantitative proteomic analysis of MEFs infected at a MOI = 1. Through comparison with noninfected MEFs cells, we identified which proteins are more upregulated and downregulated in 2022 MPXV infection compared to infection with USA‐2003 and WRAIR 7‐61. DIA resulted in the detection of 22 956 proteins in total, of which 336 proteins were identified as differentially expressed genes (DEGs) in the three MPXV strains (four biological replicates, *p* value cutoff ≤ 0.05) (Figure 3A–D). These 336 proteins included 86 viral proteins and 250 cellular proteins. All MPXV proteins have been named with the nomenclature from their VACV WR homologs.

Viral protein expression between the three strains is quite distinct, with USA 2003 having a higher protein expression compared with the other two strains (measured as Log2 Fold Change), while 2022 MPXV has the lowest amount (Figure 3E) (Table S1). Viral proteins that are more elevated in 2022 MPXV in comparison to USA 2003 include O1, Interferon Antagonist K1, and Thymidine Kinase J2. Meanwhile, WRAIR protein expression levels are intermeditae, still higher than 2022 MPXV but lower than USA 2003. A31 is one of the only proteins of interest that is notably more elevated in WRAIR rather than USA 2003.

**Figure 4:**
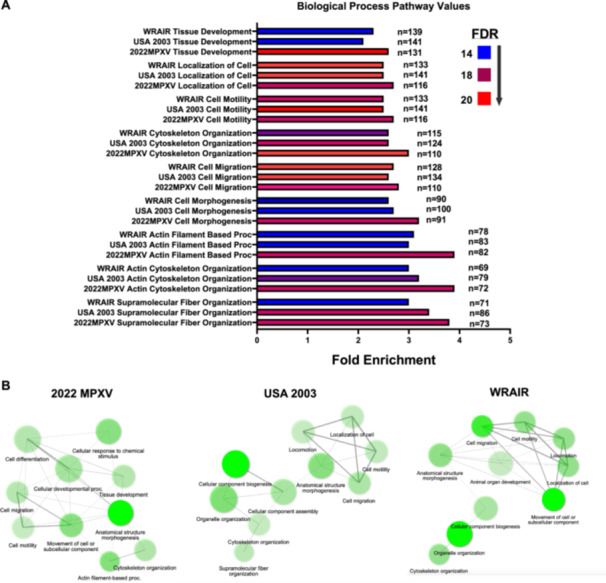
Pathway analysis indicates elevated enrichment of biological processes in 2022 MPXV infected cells when compared with USA 2003 and WRAIR. (A) Graph comparing fold enrichment of common biological process pathways produced in each MPXV strain infection. Fold enrichment indicates the number of proteins found to be associated with the indicated pathways. False discovery rate (FDR) has been colored from blue to red, with blue indicating less trustworthy and red indicating more certainty in values calculated. *N* displays the number of proteins found to be associated with the indicated pathway. (B) Network graphs displaying the most enriched biological process pathways in each strain infection in comparison to mock cells.

Among the cellular proteins, important variations were also observed. Out of 212 cellular proteins, 68 were significantly upregulated in MPXV‐infected cells and 144 cellular proteins were significantly downregulated (Table S2). Biological process pathway enrichment analysis statistically identified actin cytoskeleton and supramolecular fiber organization as the most enriched pathways. Strikingly, 2022 MPXV infection, despite being the strain with the lower effect and viral protein expression, is the strain that leads to higher enrichment of biological process in general when compared with USA 2003 and WRAIR (Figure 4).

It is worth noting that some specific proteins involved in microtubule organization are very highly upregulated during infection such as FAM110C and Beta‐Parvin. However, other proteins related to microtubules and actin formation, for example, Palladin, Actin‐binding LIM protein 1, and Actin‐filament‐associated protein 1 have their expression considerably reduced. This correlates with the increased biological process pathways depicted in Figure 4. Also of interest is that proteins with apoptotic functions are also reduced (death‐associated protein kinase 2, Caspase 12, and Programmed cell death protein 4). Additional studies will be required to investigate the importance of this strain‐specific regulation of host proteins and its relationship to the viral cycle.

### 3.3 Phospho‐Proteomic Analysis Displays Increased Phosphorylation in 2022 MPXV Infected Cells

**Figure 5:**
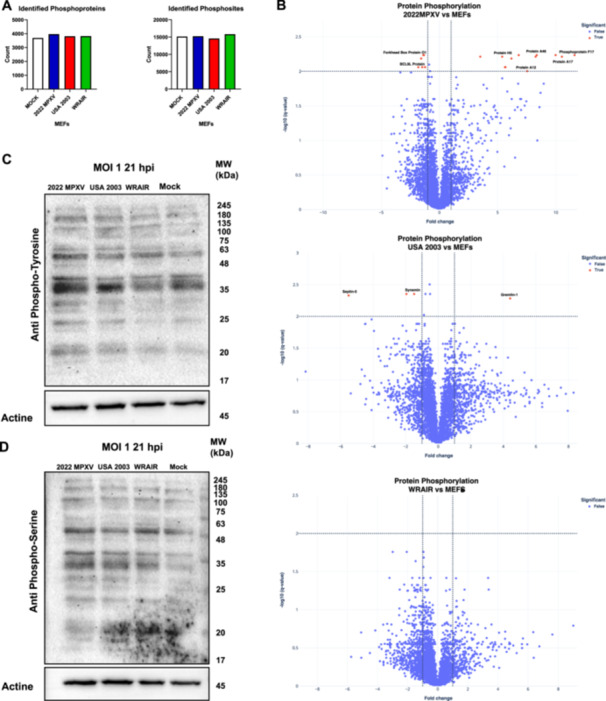
Phospho‐proteomic analysis indicated increased phosphorylation in 2022 MPXV infected cells. (A) Number of phosphoproteins and phosphosites identified in each strain infection. (B) Volcano plots of significantly altered phosphorylated proteins from Clade II MPXV infection in comparison to mock cells. (C) Western blot analysis of MPXV‐infected MEF lysates using an anti‐Tyrosine antibody. β‐Actin was used as a loading control. (D) Western blot analysis of MPXV infected MEF lysates using an anti‐Serine antibody. β‐Actin was used as a loading control.

To complement our proteomic analysis and better understand the differences in biological processes that occur in each Clade II strain infection, a phospho‐proteomic study was carried out. A total of 3697, 3973, 3818, 3828 phosphoproteins and 15 136, 15 234, 14,570, 15 833 phosphosites were identified in Mock cells, 2022 MPXV, USA 2003 and WRAIR 7‐61 infected cells, respectively (Figure 5A). Surprisingly, no significant differences were found in phosphorylated proteins in WRAIR‐infected cells, and only three cellular proteins were identified as distinctly phosphorylated in USA‐2003 infection. Septin‐5 and Synemin 1 were less phosphorylated, and Gremlin 1 was more phosphorylated. Meanwhile, 2022 MPXV had the highest amount of significantly phosphorylated proteins, with a number of these proteins being viral proteins that are known to undergo phosphorylation (Figure 5B). Statistically significant phosphorylation in viral proteins during NY 2022 infection was observed in Protein H5, Protein A12, Protein A46, Protein A17, and Phosphoprotein F17. Phosphorylated viral proteins were identified in USA 2003 and WRAIR 7‐61 infection but were considered false values due to not meeting the *q*‐value threshold (–log10 > 2) even though they were over the fold change cutoff point (Log2 > 1).

To validate these results, we did western blot analysis of MOI 1 infected cell samples at 21 hpi. Anti‐phosphorylated tyrosine and anti‐phosphorylated serine antibodies were used to visualize differences in phosphorylation in each Clade II strains when compared with noninfected cells. Through this analysis, we can observe that phosphorylated tyrosine residues are in more quantity in 2022 MPXV infection, while WRAIR has the least amount (Figure 5C). This would correlate with the phospho‐proteomic analysis. On the other hand, phosphorylated serine residues seem to be more similar between strains, though 2022 MPXV still displays the strongest bands (Figure 5D).

## 4 Discussion

**Figure 6:**
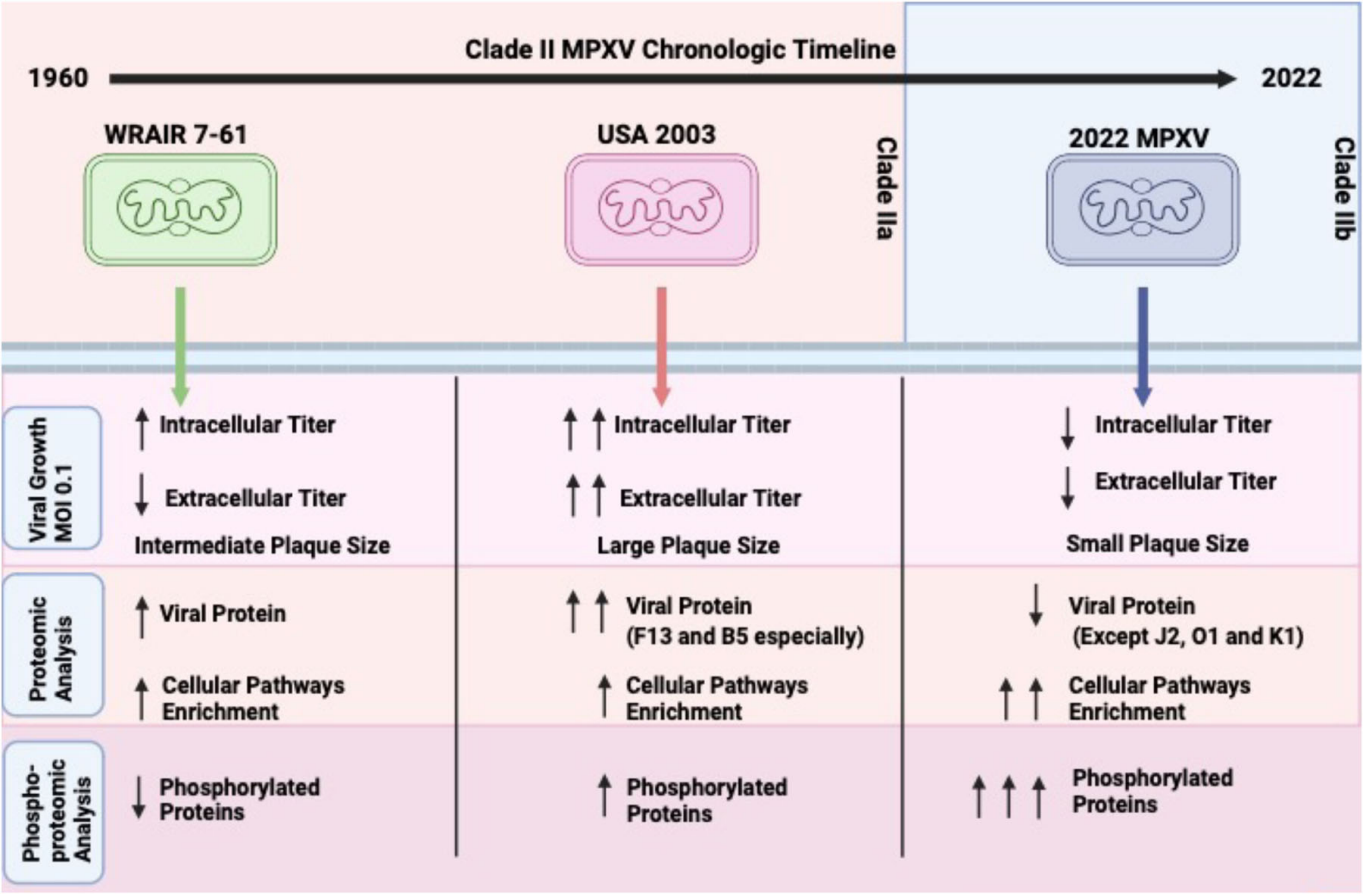
Schematic representation of differences found between Clade II MPXV strains (WRAIR 7‐61, USA 2003, and 2022 MPXV) in MEF cell infection. Created with Biorender.

The Clade IIb outbreak of MPOX is a significant worldwide public health issue that informed us of the potential danger that a more transmissible human‐to‐human transmission (HHT) MPXV could cause in the future. This is even more relevant due to recent outbreaks that led to cases of infection in vaccinated individuals [23, 31]. In addition, an uptick of Clade I cases in the Democratic Republic of Congo is a grave cause for concern [32]. The specific viral mechanisms that led to lineage B.1 MPXV increased dissemination are still unknown. Through analysis of previous Clade II strains in conjunction with the 2022 MPXV strain, our aim is to elucidate the differences between them that could have being involved in the higher magnitude of the Clade IIb outbreak of MPOX. WRAIR 7‐61 is our oldest Clade II reference strain, derived from an isolate obtained from infected Cynomolgus monkeys with Western Africa origins [33]. While there have been reports of HTH of Clade I strain in Central Africa, there have not been any records of this type of transmission in Western Africa. Meanwhile, USA 2003 is a representative MPXV isolate of the strain responsible for the 2003 Wisconsin outbreak. This outbreak originated from MPXV‐infected Ghana rodents that came into contact with prairie dogs. Human cases were hypothesized to be mainly from close contact with infected prairie dogs, with no HHT [21, 34, 35]. It is of interest to study the main difference between Clade IIa and IIb, which is the higher transmissibility characteristics in Clade IIb among humans instead of in animals. As mentioned before, the Lineage B.1 MPXV reference strain that we used in these studies is an isolate obtained from a patient during the 2022 MPOX outbreak. Our comparative analysis was undertaken primarily using MEF cells. Since rodents are hypothesized to be one of the natural reservoirs of MPXV, it is important to analyze Clade II strains in rodent models [36]. In addition, further studies in human cells and reservoir‐matched rodents need to be undertaken to better understand the different phenotypes of these three representative MPXV strains (Figure 6).

To study virus production after several rounds of infection, we conducted experiments at low multiplicity infection, in which we can highlight key differences between the MPXV Clade II strains. Intracellular and extracellular virus titer was quantified to have an approximation in the amount of MVs and EVs, respectively. 2022 MPXV displayed slower viral growth in comparison to USA 2003 and WRAIR 7‐61 when measuring intracellular titers. We speculate that the more gradual infection by Lineage B.1 MPXV could be an important reason for the worldwide outbreak of the virus. Less virulent viruses are more prone to causing contagion due to lessened mortality rates and symptoms, with a recent example being SARS‐CoV‐2 compared with MERS and the first SARS‐CoV [37]. This difference in titer and mortality is even more pronounced when comparing Lineage B.1 MPXV to Clade I MPXV, such as in the study of Americo et al. [38]. To investigate the behavior of 2022 MPXV in human cells compared with WR, another poxvirus reference, we quantified viral replication in a human A549 cell line. The 2022 MPXV viral titers in human cells were observed to be increased in comparison to those in MEF cells. Nevertheless, 2022 MPXV replicates at a significantly lower level than VACV WR in both cell lines. It is noteworthy that the viral titers of WR remain largely unchanged after infection of human or mouse cell lines. Our results clearly demonstrated that 2022 MPXV replicates at a much faster rate in these human cells when compared with MEFs, suggesting that the strain has better adapted to the human host.

Statistically significant differences could be observed when comparing extracellular titers of USA 2003 with 2022 MPXV and WRAIR 7‐61. Previously, this EV increase was also observed in a monkey‐derived cell line after infection with USA 2003 [38]. A pronounced difference in MV/EV ratio is a hypothesis for the spread of lineage B.1 MPXV [39]. Transmission of Clade IIb MPXV is thought to be mainly occurring via a primary localized rash, instead of a general disseminated infection that would correlate with a distinct viral cycle programing. Previously, MPOX caused a generalized monomorphic pustular rash in patients, whereas this Clade IIb strain causes an ulcerative genital rash [40]. This characteristic of primary localized lesions would correlate with our findings that 2022 MPXV, in contrast to USA 2003, produces a very high proportion of intracellular associated titers, leading to a much higher transmissibility. Another important factor in the transmissibility of MPOX is the gradual decline in the population of individuals with natural immunity to poxviruses and who have been vaccinated against smallpox [1][2]. Studies have shown that individuals vaccinated against smallpox continue to produce antibodies against Clade II MPXV [41]. This demonstrates that poxvirus vaccines are highly effective and that efforts must be made to vaccinate young and vulnerable populations, such as the MSM community and Central African populations, to successfully eradicate this disease.

Proteomic analysis revealed a great amount of information regarding viral protein expression in the different MPXV Clade II strains. First, 2022 MPXV infection does not lead to as much viral protein production. This would align with 2022 MPXV slower growth, but there is not a clear correlation that intermediate and late viral proteins are reduced in comparison to early proteins. For example, early proteins C1, D7, and F3 are all in very low quantity compared to USA 2003 and WRAIR. On the other hand, late proteins such as A4, A29, and H7 are similar to the other two strains. Further study indicated that 2022 MPXV infection only produced three viral proteins in higher abundance than USA 2003, which were J2, K1, and O1. J2 is a thymidine kinase, which is a protein of interest due to its role in viral DNA replication. Experiments analyzing its effect in a thymidine kinase‐negative phenotype of VACV informed us that its removal led to a decrease in virulence [42]. Second, K1 is a host range gene that has been previously demonstrated to be important in human cell replication along with C7 [43]. A hypothesis could be that its increment could have led to more efficient replication in human cells of Lineage B.1 MPXV. Lastly, O1 proteins specific function is unknown, but it has been attributed to sustaining the upregulation of extracellular signal‐regulated kinase 1/2 pathway and promoting viral virulence [44].

Other proteins of interest are F13 and B5, which are typically associated with EVs [45, 46]. As expected, USA 2003 is the strain that has the highest presence of these proteins, once again validating the extracellular titer measured. While this could be expected due to most protein values being higher, these two proteins are two of the highest values found in USA 2003 infection. This informs us that elevated production of EVs may not be as important in order for efficient HHT, which is the case of Lineage B.1 MPXV.

Results obtained from the proteomic analysis of cellular proteins in virus‐infected cells informed us of possible host cell mechanisms that MPXV is using to its advantage. 2022 MPXV infection caused the highest amount of enrichment in all of the cellular pathways analyzed. This could indicate that Lineage B.1 MPXV is using the cellular machinery more than USA 2003 and WRAIR. It could also be that the more rapid growth of these two strains affected the number of cellular proteins identified by inducing a more profound host cell protein shut‐off. Notably, increased pathways are related to actin and cytoskeleton organization. This is relevant because VACV and MPXV are known to use the cellular cytoskeleton in its dissemination, for example, using actin tails [8, 47]. Specific proteins that were altered significantly related to cytoskeleton organization have been identified. For example, FAM100C and Beta‐Parvin expression is greatly upregulated, while Palladin, Actin binding LIM protein 1, and Actin‐filament associated protein 1 expression is downregulated.

Phosphorylation plays an important part in viral infection, as seen in a number of relevant viruses such as Flaviviruses, Hepaciviruses, and Coronaviruses. Viruses are known to exploit numerous kinases and cellular pathways during their viral cycle [48]. VACV presents numerous viral proteins that undergo phosphorylation which would be important for its viral cycle. These viral proteins for example are F17, A14, and A17 [49]. Recently, a multi‐omics characterization of 2022 MPXV infection demonstrated the importance of phosphorylation during infection [50]. In our present study, we compare the phosphorylation levels between 2022, USA 2003, and WRAIR MPXV strains demonstrating a significantly higher increase in phosphorylation of lineage B.1 MPXV compared with the previous Clade II strains. These changes may be due to a difference in the dynamics of the viral cycle observed between the strains. Concerning the observed differences in proteins involved in phosphorylation pathways, the FOXO1 protein, which is critical for the OXPHOS pathway and energy metabolism, showed a significantly reduced phosphorylation level exclusively after infection with the 2022 MPX virus. This finding supports the hypothesis that the OXPHOS pathway plays a critical role in viral replication, as previously reported [51].

In vitro experiments are an important tool for understanding the viral dynamics that can lead to outbreaks. It is crucial to uncover differences in the viral cycle in the natural reservoirs of the disease, in this case, rodents, that may lead to its higher transmissibility among humans. However, in vitro results are subject to a number of limitations with respect to the many factors that may affect viral growth in human patients. Interactions with other drugs, previous exposure to the virus and co‐morbidities can severely affect the outcome of MPOX in an individual [52]. The latter is particularly relevant considering that people with HIV are over‐represented in current MPOX cases [22]. Although this limitation exists in the in vitro studies, further techniques such as the use of organoids could be useful to improve our knowledge in a more physiological way [53]. It may also be interesting in the future to use different pharmaceuticals and approved MPOX vaccines to improve in vitro studies. This may help us to obtain more clinically relevant results regarding the amounts of these treatments needed to reduce MPXV replication and the effects they may have on future MPXV strains.

The present manuscript findings highlight significant differences between Lineage B.1 MPXV and previous Clade II strains in its viral growth and protein expression. This is even more relevant due to the increasing severity of the Clade I MPXV outbreak in the DRC, with the worry that this deadlier Clade could develop the transmissibility characteristics of Lineage B.1 MPXV. Further research will be necessary to better understand the significance of these differences in the context of the novel transmission mechanisms observed in the Clade IIb outbreak to prevent MPOX outbreaks in the near future.

## Author Contributions

Susana Guerra contributed to conception and design of the study. Joseph Patrick McGrail performed the experiments. Joseph Patrick McGrail performed the statistical analysis. Alberto Paniz Mondolfi, Juan David Ramírez, Susana Guerra, Mari Paz Sanchez‐Seco, Adolfo García‐Sastre, Santiago Vidal and Gustavo Palacios provided reagents. Joseph Patrick McGrail and Susana Guerra wrote the manuscript. All authors contributed to final manuscript revision, read and approved the submitted version.

## Conflicts of Interest

The A.G.‐S. laboratory has received research support from GSK, Pfizer, Senhwa Biosciences, Kenall Manufacturing, Blade Therapeutics, Avimex, Johnson & Johnson, Dynavax, 7Hills Pharma, Pharmamar, ImmunityBio, Accurius, Nanocomposix, Hexamer, N‐fold LLC, Model Medicines, Atea Pharma, Applied Biological Laboratories and Merck, outside of the reported work. A.G.‐S. has consulting agreements for the following companies involving cash and/or stock: Castlevax, Amovir, Vivaldi Biosciences, Contrafect, 7Hills Pharma, Avimex, Pagoda, Accurius, Esperovax, Applied Biological Laboratories, Pharmamar, CureLab Oncology, CureLab Veterinary, Synairgen, Paratus, Pfizer and Prosetta, outside of the reported work. A.G.‐S. has been an invited speaker in meeting events organized by Seqirus, Janssen, Abbott, Astrazeneca, and Novavax. A.G.‐S. is inventor on patents and patent applications on the use of antivirals and vaccines for the treatment and prevention of virus infections and cancer, owned by the Icahn School of Medicine at Mount Sinai, New York, outside of the reported work. The remaining authors declare no conflict of interest.

## Supporting information

Supporting information.

Supporting information.

Supporting information.

Supporting information.

Supporting information.

Supporting information.

## Data Availability

The data that support the findings of this study are available from the corresponding author upon reasonable request.
