# Supplementary figures and images for: Comparative Analysis of 2022 Outbreak MPXV and Previous Clade II MPXV
Source: J Med Virol. 2024 Oct 28;96(11):e70023. doi: 10.1002/jmv.70023 (PMC11600476; doi:10.1002/jmv.70023)

A

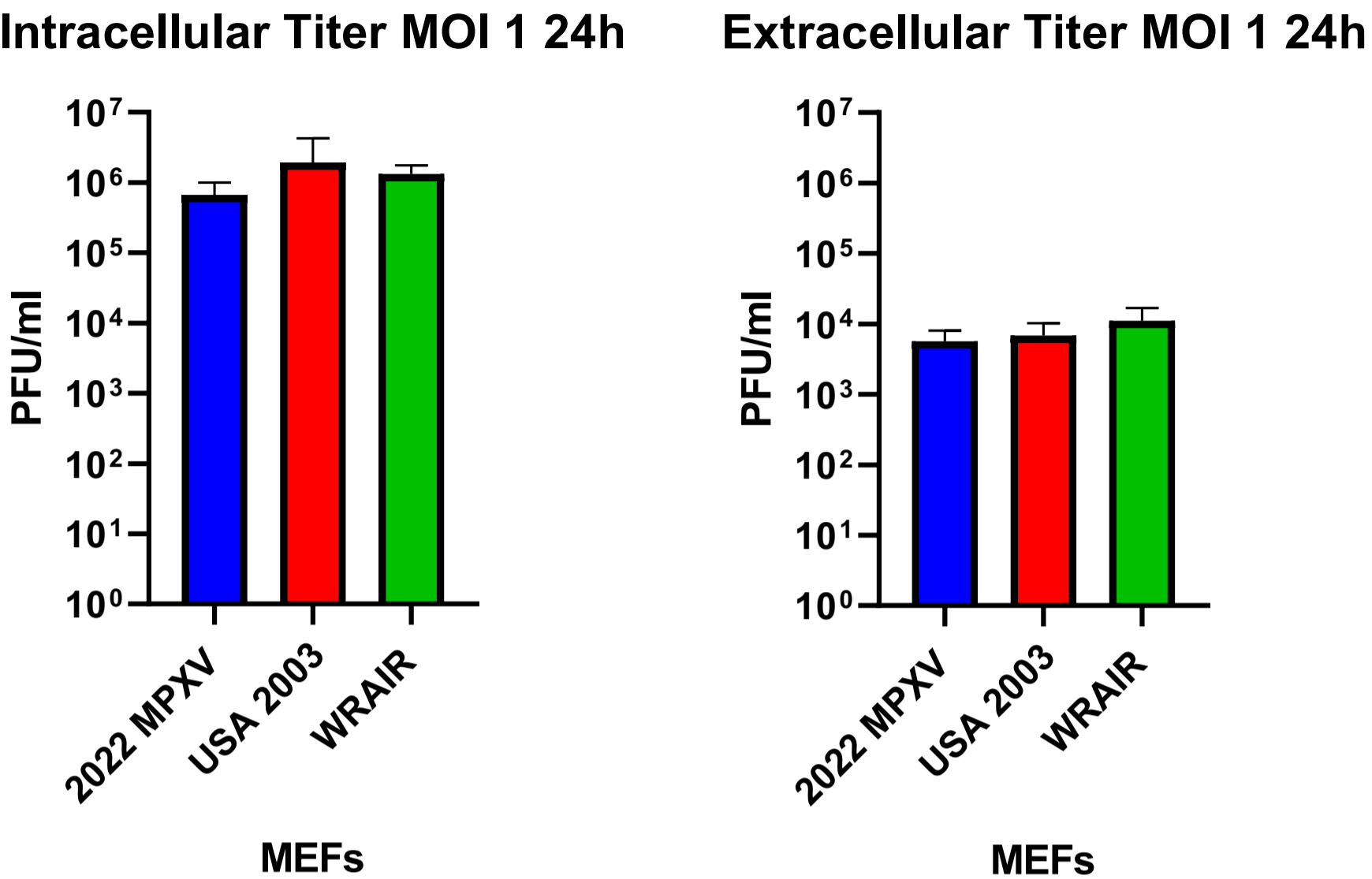

B

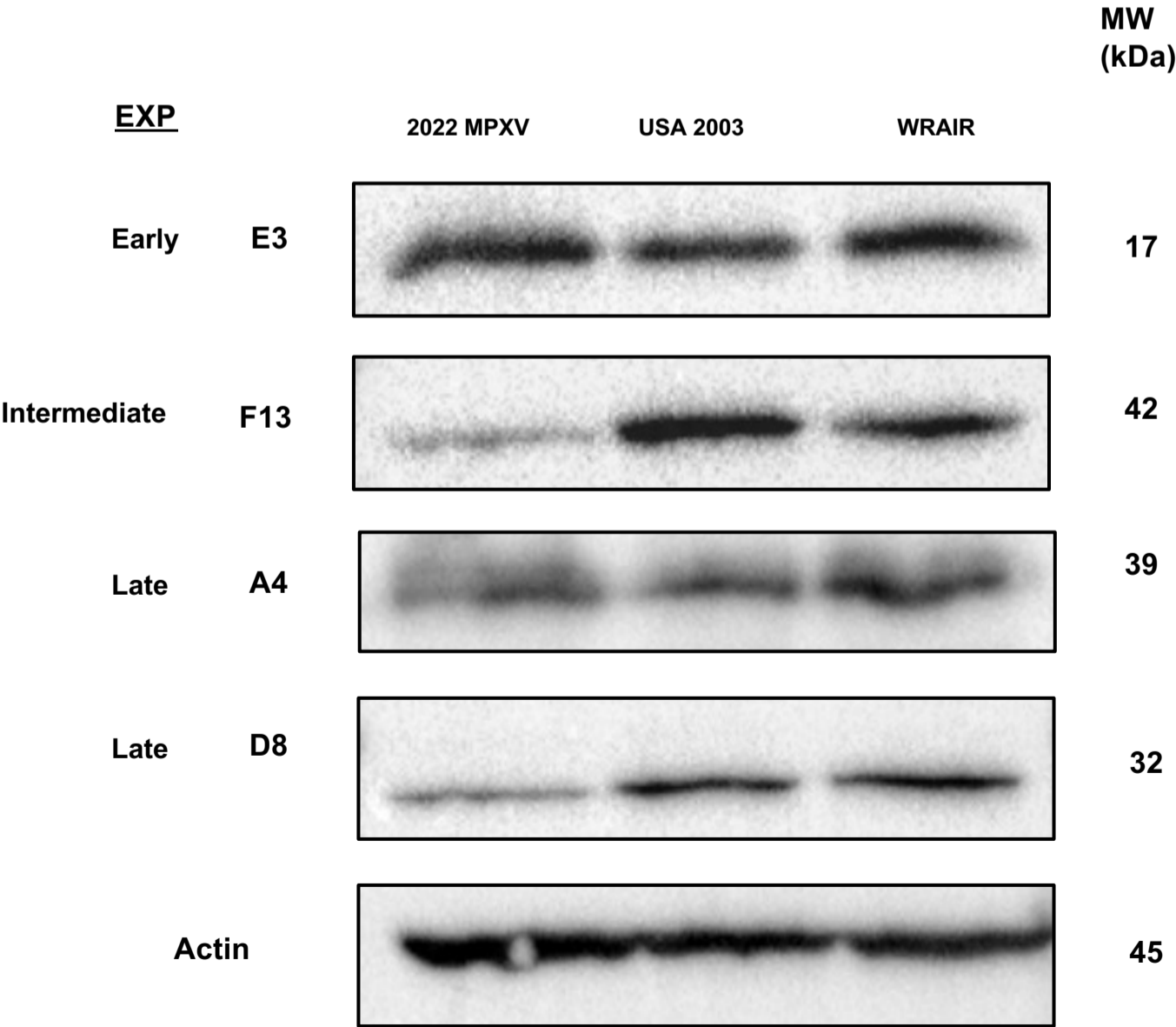

Supplement: Supplementary file 1 — Supporting information. [file JMV-96-e70023-s003.pdf]
