# Supplementary material for: Comparative Analysis of 2022 Outbreak MPXV and Previous Clade II MPXV
Source: J Med Virol. 2024 Oct 28;96(11):e70023. doi: 10.1002/jmv.70023 (PMC11600476; doi:10.1002/jmv.70023)
Supplement: Supplementary file 2 — Supporting information. [file JMV-96-e70023-s005.docx]

**Supplementary Figure 1.** (A) MEFs were infected with the three MPXV strain (MOI 1), at 24 hpi, infectious viral particles from supernatants (extracellular virus) and cell extracts (intracellular virus) were titrated by plaque assay. Mean ± SD values from four independent experiments are represented. (B) MEFs were infected with the three MPXV strain (MOI 1) and, at 24hpi, equal amounts of proteins from cell extracts were analyzed by Western Blot. Specific antibodies for VACV early protein E3, intermediate protein F13 and late proteins D8 and A4 were used. β-Actin was used as loading control. Molecular weights (MW) in kilodaltons (kDa) are indicated, based on protein standards. Means ± the SD from three independent experiments is represented. *, P < 0.05; **, P < 0.01; ***, P < 0.005; ****, P < 0.0001.
