# Supplementary material for: Comparative Analysis of 2022 Outbreak MPXV and Previous Clade II MPXV
Source: J Med Virol. 2024 Oct 28;96(11):e70023. doi: 10.1002/jmv.70023 (PMC11600476; doi:10.1002/jmv.70023)
Supplement: Supplementary file 3 — Supporting information. [file JMV-96-e70023-s006.docx]

**Supplementary Table 1.** Viral protein signature of MPXV MOI 1 infected MEF lysates at 21 hours post-infection. Viral proteins using VACV WR homologues nomenclature. Protein ID, encoding gene name, log2-fold change (FC) (MPXV infected MEF versus MEF Mock) of levels of each protein, and the statistical significance (−log P value) of each Clade II strain are listed.
