# Supplementary material for: Comparative Analysis of 2022 Outbreak MPXV and Previous Clade II MPXV
Source: J Med Virol. 2024 Oct 28;96(11):e70023. doi: 10.1002/jmv.70023 (PMC11600476; doi:10.1002/jmv.70023)
Supplement: Supplementary file 4 — Supporting information. [file JMV-96-e70023-s004.docx]

**Supplementary Table 2.** Cellular protein signature of MPXV MOI 1 infected MEF lysates at 21 hours post-infection. Protein ID, encoding gene name, log2-fold change (FC) (MPXV infected MEF versus MEF Mock) of levels of each protein, and statistical significance (−log P value) of each Clade II strain are listed.
