# Supplementary material for: Comparative Analysis of 2022 Outbreak MPXV and Previous Clade II MPXV
Source: J Med Virol. 2024 Oct 28;96(11):e70023. doi: 10.1002/jmv.70023 (PMC11600476; doi:10.1002/jmv.70023)
Supplement: Supplementary file 5 — Supporting information. [file JMV-96-e70023-s002.docx]

**Supplementary table 1**. Viral protein signature of MPXV infected cellular lysates from MEFs ^a^.

| Protein ID | EXP | OPG | Gene | 2022 MPXV  Log2FC | 2022 MPXV  -LogP | USA  2003  Log 2FC | USA  2003  -LogP | 7-61  Log2FC | 7-61    -LogP |
| --- | --- | --- | --- | --- | --- | --- | --- | --- | --- |
| P0DTM9 | E | OPG  001 | C23L/  B29R | 5.3 | 1.6 | 7.9 | 2.3 | 6.9 | 2.0 |
| P0DTN0 | E | OPG  002 | B28R | 6.8 | 3.7 | 8.7 | 4.1 | 8.7 | 3.9 |
| A0A7H0DNH0 | L | OPG  003 | C19L/  B25R | 3.3 | 2.5 | 3.9 | 3.0 | 3.9 | 2.8 |
| A0A7H0DNG7 | E | OPG  005 | C16L | 6.3 | 3.7 | 7.4 | 4.1 | 7.4 | 4.0 |
| A0A7H0DMZ6 | E | OPG  019 | C11R | 3.8 | 2.7 | 4.6 | 3.2 | 3.3 | 2.5 |
| A0A7H0DMZ8 | E | OPG  022 | OPG022 | 5.0 | 1.5 | 8.0 | 2.4 | 7.6 | 2.2 |
| A0A7H0DMZ9 | E | OPG  023 | MPXV  gp011 | 3.1 | 1.3 | 3.9 | 1.8 | 4.4 | 1.9 |
| A0A7H0DN02 | E | OPG  027 | C7L | 5.5 | 4.3 | 7.0 | 4.9 | 6.5 | 4.4 |
| A0A7H0DN07 | E | OPG  034 | C1L | 2.2 | 3.0 | 3.9 | 3.9 | 2.9 | 3.5 |
| P0DTN4 | E | OPG  035 | N1L | 7.7 | 5.5 | 8.3 | 5.8 | 8.1 | 5.4 |
| A0A7H0DN09 | E | OPG  036 | N2L | 4.7 | 3.1 | 5.4 | 3.5 | 5.4 | 3.4 |
| A0A7H0DN11 | E | OPG  038 | M2L | 5.7 | 3.7 | 8.1 | 4.6 | 7.1 | 4.2 |
| A0A7H0DN12 | E | OPG  039 | K1L | 5.8 | 4.5 | 5.7 | 4.6 | 5.0 | 4.2 |
| A0A7H0DN15 | I | OPG  042 | K4L | 4.5 | 3.3 | 7.0 | 4.1 | 5.8 | 3.6 |
| A0A7H0DN16 | E | OPG  043 | C5L,  K5L | 5.1 | 2.1 | 6.3 | 2.6 | 5.3 | 2.3 |
| A0A7H0DN18 | E | OPG  045 | F1L | 3.4 | 2.8 | 6.1 | 3.7 | 5.7 | 3.5 |
| A0A7H0DN19 | E | OPG  046 | F2L | 7.3 | 3.7 | 8.3 | 4.0 | 8.5 | 3.9 |
| A0A7H0DN20 | E | OPG  047 | F3L | 1.9 | 1.4 | 3.9 | 2.8 | 3.7 | 2.6 |
| A0A7H0DN23 | E | OPG  050 | F6L | 3.1 | 3.3 | 5.5 | 4.3 | 4.1 | 3.6 |
| A0A7H0DN24 | E | OPG  051 | F7L | 4.0 | 2.7 | 5.4 | 3.3 | 5.4 | 3.2 |
| A0A7H0DN25 | E | OPG  052 | F8L | 7.1 | 4.1 | 7.8 | 4.4 | 7.5 | 4.1 |
| A0A7H0DN28 | L | OPG  055 | F11L | 5.4 | 4.7 | 7.1 | 5.1 | 5.9 | 4.5 |
| A0A7H0DN30 | I | OPG  057 | F13L | 3.1 | 1.5 | 6.5 | 2.8 | 5.2 | 2.3 |
| A0A7H0DN35 | L | OPG  062 | F17R | 5.9 | 3.5 | 7.6 | 4.0 | 6.6 | 3.5 |
| A0A7H0DN36 | E | OPG  063 | E1L | 4.5 | 3.6 | 5.5 | 4.1 | 4.7 | 3.6 |
| A0A7H0DN38 | E | OPG  065 | E3L | 9.8 | 5.2 | 10.1 | 5.2 | 10.2 | 4.8 |
| A0A7H0DN39 | E | OPG  066 | E4L | 6.4 | 4.0 | 7.9 | 4.5 | 6.9 | 4.0 |
| A0A7H0DN42 | I | OPG  069 | E7R | 6.2 | 3.7 | 6.8 | 4.0 | 6.6 | 3.8 |
| A0A7H0DN44 | E | OPG  071 | E9L | 5.3 | 3.0 | 5.8 | 3.3 | 5.3 | 3.0 |
| A0A7H0DN46 | I | OPG  073 | E11L | 5.3 | 3.8 | 7.2 | 4.4 | 6.1 | 4.0 |
| A0A7H0DN47 | E | OPG  074 | O1L | 6.1 | 3.4 | 5.8 | 3.5 | 4.9 | 3.1 |
| A0A7H0DN48 | I | OPG  075 | O2L | 5.2 | 1.4 | 6.1 | 1.7 | 6.1 | 1.7 |
| A0A7H0DN56 | I | OPG  084 | I8R | 4.3 | 3.5 | 5.5 | 4.1 | 4.6 | 3.4 |
| A0A7H0DN57 | L | OPG  085 | G1L | 3.9 | 3.6 | 5.5 | 4.2 | 4.4 | 3.5 |
| M1L9M3 | E | OPG  087 | G2R | 3.3 | 3.0 | 5.8 | 4.0 | 4.9 | 3.6 |
| M1L502 | L | OPG  088 | G4L | 5.0 | 3.9 | 6.7 | 4.8 | 5.8 | 4.0 |
| A0A7H0DN61 | E | OPG  089 | G5R | 2.9 | 1.5 | 3.4 | 1.8 | 3.2 | 1.7 |
| A0A7H0DN62 | E | OPG  090 | G5.5R | 4.6 | 2.8 | 6.3 | 4.0 | 5.9 | 3.9 |
| A0A7H0DN68 | E | OPG  096 | L2R | 3.2 | 3.5 | 4.9 | 4.1 | 3.8 | 4.1 |
| A0A7H0DN69 | L | OPG  097 | L3L  /F4 | 3.9 | 3.7 | 5.8 | 4.4 | 4.7 | 3.6 |
| M1LBP0 | L | OPG  099 | L1R  /L5R | 1.5 | 1.5 | 4.2 | 3.1 | 2.8 | 2.3 |
| A0A7H0DN73 | E | OPG  101 | J2R | 7.6 | 3.6 | 6.4 | 3.5 | 7.7 | 3.6 |
| A0A7H0DN80 | L | OPG  108 | H3L | 5.5 | 1.4 | 7.0 | 1.8 | 5.9 | 1.5 |
| A0A7H0DN81 | L | OPG  109 | H4L | 4.5 | 2.5 | 5.6 | 3.0 | 4.7 | 2.6 |
| A0A7H0DN82 | CE | OPG  110 | H5R | 8.1 | 4.5 | 8.7 | 4.8 | 8.0 | 4.4 |
| A0A7H0DN84 | L | OPG  112 | H7R | 6.1 | 4.5 | 6.5 | 4.8 | 6.3 | 4.4 |
| M1KJ15 | L | OPG  114 | D2L | 4.3 | 4.0 | 6.5 | 4.7 | 5.5 | 4.3 |
| A0A7H0DN90 | L | OPG  118 | D6R | 3.4 | 2.5 | 4.1 | 3.1 | 3.5 | 2.6 |
| A0A7H0DN91 | E | OPG  119 | D7R | 3.0 | 2.9 | 5.0 | 3.8 | 4.4 | 3.4 |
| A0A7H0DN96 | E | OPG  124 | D12L | 5.3 | 1.7 | 5.9 | 2.0 | 5.1 | 1.7 |
| M1LLA2 | I | OPG  126 | A1L | 3.3 | 1.5 | 5.4 | 2.4 | 4.6 | 2.0 |
| A0A7H0DN99 | I | OPG  127 | A2L | 3.0 | 3.2 | 4.3 | 4.0 | 3.7 | 3.5 |
| A0A7H0DNA2 | L | OPG  130 | A4L | 8.0 | 4.0 | 9.6 | 4.5 | 8.5 | 4.0 |
| A0A7H0DNA3 | E | OPG  131 | A5L | 4.9 | 3.7 | 6.5 | 4.3 | 5.6 | 3.8 |
| A0A7H0DNA5 | E | OPG  133 | A7L | 4.5 | 1.6 | 5.6 | 2.1 | 4.7 | 1.8 |
| A0A7H0DNA6 | I | OPG  134 | A8R | 2.7 | 3.4 | 4.1 | 5.0 | 3.9 | 4.8 |
| M1L543 | L | OPG  138 | A12L | 5.3 | 1.7 | 6.0 | 2.0 | 5.1 | 1.7 |
| P0DTN1 | L | OPG  139 | A13L | 5.0 | 3.7 | 7.5 | 4.3 | 6.0 | 3.6 |
| A0A7H0DNB2 | L | OPG  140 | A14L | 3.2 | 2.1 | 6.0 | 3.3 | 4.6 | 2.7 |
| A0A7H0DNB4 | L | OPG  142 | A15L | 3.4 | 3.4 | 5.9 | 4.4 | 5.4 | 3.9 |
| A0A7H0DNB6 | L | OPG  144 | A17L | 8.0 | 3.4 | 9.5 | 3.9 | 8.6 | 3.6 |
| A0A7H0DNB7 | L | OPG  145 | A18R | 4.8 | 2.9 | 7.0 | 3.7 | 6.4 | 3.4 |
| A0A7H0DNC1 | I | OPG  149 | A22R | 2.8 | 2.9 | 3.9 | 3.4 | 3.3 | 3.0 |
| A0A7H0DNC4 | L | OPG  153 | A26R | 5.3 | 1.8 | 7.1 | 2.5 | 6.0 | 2.1 |
| P0DTN3 | I | OPG  154 | A27L | 5.4 | 1.7 | 7.2 | 2.3 | 6.0 | 1.9 |
| A0A7H0DNC7 | L | OPG  156 | A29L | 6.4 | 2.2 | 7.4 | 2.6 | 6.9 | 2.4 |
| A0A7H0DNC8 | L | OPG  157 | A30L | 3.7 | 2.9 | 5.2 | 3.7 | 4.4 | 3.2 |
| A0A7H0DND0 | E | OPG  159 | A31R | 5.5 | 3.3 | 7.3 | 3.9 | 8.5 | 3.2 |
| A0A7H0DND5 | E | OPG  164 | A36R | 4.8 | 2.9 | 6.8 | 3.6 | 6.4 | 3.4 |
| A0A7H0DND6 | E | OPG  165 | A37R | 4.7 | 2.7 | 6.2 | 3.3 | 5.0 | 2.9 |
| A0A7H0DND8 | E | OPG  170 | A41L | 3.8 | 3.7 | 6.6 | 4.6 | 4.9 | 4.0 |
| A0A7H0DNE1 | E | OPG  173 | OPG173 | 4.7 | 4.2 | 5.5 | 4.9 | 5.0 | 4.1 |
| A0A7H0DNE2 | E | OPG  174 | A44L | 4.9 | 3.9 | 5.8 | 4.3 | 5.8 | 4.1 |
| A0A7H0DNE4 | E | OPG  176 | A46R | 7.6 | 5.2 | 8.2 | 5.2 | 7.7 | 4.8 |
| A0A7H0DNE6 | E | OPG  180 | A50R | 6.0 | 3.7 | 7.9 | 4.3 | 6.8 | 3.9 |
| A0A7H0DNE7 | E | OPG  181 | A51R | 5.5 | 2.0 | 7.5 | 2.7 | 6.5 | 2.3 |
| A0A7H0DNE9 | E | OPG  187 | B1R | 5.5 | 2.6 | 6.6 | 3.1 | 6.1 | 2.8 |
| A0A7H0DNF0 | E | OPG  188 | B2R | 6.8 | 3.6 | 6.9 | 3.9 | 7.6 | 3.9 |
| P0DTN2 | E | OPG  190 | B5R | 5.4 | 2.7 | 8.7 | 3.6 | 7.5 | 3.2 |
| A0A7H0DNF5 | E | OPG  193 | B8R | 5.8 | 4.5 | 8.7 | 4.9 | 8.7 | 4.6 |
| A0A7H0DNF8 | E | OPG  198 | B12R | 4.5 | 3.7 | 5.7 | 4.6 | 5.6 | 4.3 |
| A0A7H0DNF9 | E | OPG  199 | B13R | 6.7 | 1.6 | 6.9 | 1.7 | 6.7 | 1.6 |
| A0A7H0DNG0 | E | OPG  200 | B14R | 4.7 | 4.0 | 5.5 | 4.5 | 5.6 | 4.3 |
| A0A7H0DNG2 | E | OPG  204 | B18R | 7.0 | 3.7 | 8.8 | 4.2 | 7.6 | 3.8 |
| A0A7H0DNG4 | E | OPG  205 | C12L | 4.2 | 2.1 | 4.9 | 2.5 | 5.4 | 2.5 |
| A0A7H0DNG5 | E | OPG  209 | C13L | 3.9 | 2.3 | 6.0 | 3.1 | 5.9 | 3.0 |

**a** Viral protein signature of MPXV MOI 1 infected MEF lysates at 21 hours post-infection. Viral proteins are using VACV WR homologues nomenclature. Protein ID, protein name, gene name, log2-fold change (FC) (MPXV infected MEF versus MEF Mock) of the levels of each protein, and statistical significance (−log P value) of each Clade II strain are listed.
