# Supplementary material for: Comparative Analysis of 2022 Outbreak MPXV and Previous Clade II MPXV
Source: J Med Virol. 2024 Oct 28;96(11):e70023. doi: 10.1002/jmv.70023 (PMC11600476; doi:10.1002/jmv.70023)
Supplement: Supplementary file 6 — Supporting information. [file JMV-96-e70023-s001.docx]

**Supplementary table 2**. Cellular protein signature of MPXV infected cellular lysates from MEFs ^a^.

| Protein  ID | Gene | MPXV  2022  Log2 FC | MPXV  2022  -LogP | USA  2003  Log2  FC | USA  2003  -LogP | 7-61  Log2  FC | 7-61  -LogP |
| --- | --- | --- | --- | --- | --- | --- | --- |
| Q6BCL1 | Pram1 | 5.3 | 3.9 | 6.7 | 4.6 | 5.8 | 4.1 |
| Q8VE94 | **Fam110c** | 4.5 | 2.2 | 4.3 | 2.3 | 4.6 | 2.3 |
| Q7TPS0 | Rps6ka6 | 4.4 | 3.9 | 3.3 | 3.4 | 3.3 | 3.6 |
| P52624 | Upp1 | 4.3 | 2.1 | 3.9 | 2.2 | 3.7 | 2.0 |
| Q8R3P0 | Aspa | 4.0 | 3.0 | 4.1 | 3.2 | 4.4 | 3.2 |
| Q62266 | Sprr1a | 3.5 | 5.2 | 2.3 | 4.0 | 3.4 | 4.6 |
| P22437 | Ptgs1 | 3.4 | 1.5 | 3.9 | 1.9 | 3.2 | 1.5 |
| Q8R5M8 | Cadm1 | 3.4 | 1.9 | 3.2 | 2.0 | 4.0 | 2.2 |
| Q9WVB0 | Rbpms | 3.2 | 1.6 | 3.8 | 2.7 | 4.4 | 2.9 |
| A2A8L5 | Ptprf | 2.9 | 1.8 | 3.1 | 2.1 | 3.1 | 2.0 |
| Q9CXI0 | Coq5 | 2.9 | 2.2 | 3.1 | 2.8 | 3.5 | 2.9 |
| Q6GQW0 | Abtb3 | 2.8 | 1.6 | 2.7 | 1.7 | 2.7 | 1.7 |
| P52927 | Hmga2 | 2.8 | 3.7 | 2.6 | 3.9 | 2.7 | 3.9 |
| Q05769 | Ptgs2 | 2.7 | 5.1 | 3.6 | 4.3 | 3.4 | 4.4 |
| Q60695 | Rgl1 | 2.7 | 1.7 | 2.5 | 1.7 | 2.6 | 1.7 |
| Q9CYH2 | Prxl2a | 2.7 | 3.0 | 3.3 | 3.6 | 3.3 | 3.4 |
| Q9Z183 | Padi4 | 2.7 | 2.5 | 2.0 | 2.2 | 2.9 | 2.5 |
| Q9ES46 | **Parvb** | 2.6 | 1.6 | 2.3 | 1.6 | 2.6 | 1.7 |
| P82343 | Renbp | 2.5 | 1.6 | 3.2 | 2.1 | 3.0 | 1.9 |
| Q61490 | Alcam | 2.5 | 3.9 | 3.3 | 4.9 | 3.3 | 4.4 |
| P12388 | Serpinb2 | 2.4 | 3.0 | 1.7 | 2.2 | 1.9 | 2.4 |
| P08103 | Hck | 2.4 | 2.1 | 2.2 | 2.1 | 2.6 | 2.3 |
| Q60765 | Atf3 | 2.2 | 1.4 | 2.4 | 1.6 | 2.2 | 1.4 |
| Q05915 | Gch1 | 2.1 | 2.0 | 2.3 | 2.4 | 2.0 | 2.0 |
| P50615 | Btg3 | 2.0 | 2.3 | 1.4 | 1.9 | 1.5 | 1.8 |
| P28650 | Adss1 | 2.0 | 1.7 | 1.8 | 1.7 | 2.1 | 1.9 |
| O89114 | Dnajb5 | 1.9 | 3.0 | 1.1 | 2.1 | 1.2 | 2.4 |
| Q9CZJ1 | Utp11 | 1.9 | 2.9 | 1.3 | 1.7 | 2.1 | 2.9 |
| P48755 | Fosl1 | 1.8 | 2.9 | 2.2 | 3.5 | 2.0 | 3.0 |
| Q9CQT7 | Desi1 | 1.7 | 2.7 | 1.2 | 3.2 | 1.2 | 3.1 |
| Q60651 | Klra4 | 1.7 | 1.4 | 2.4 | 3.1 | 2.1 | 2.7 |
| P06837 | Gap43 | 1.7 | 1.4 | 2.3 | 1.9 | 2.3 | 1.8 |
| Q61739 | Itga6 | 1.7 | 2.2 | 2.3 | 2.9 | 2.2 | 2.8 |
| Q62469 | Itga2 | 1.7 | 2.5 | 2.3 | 3.1 | 1.4 | 2.1 |
| P17095 | Hmga1 | 1.6 | 3.4 | 1.2 | 4.4 | 1.5 | 4.1 |
| Q9Z1B3 | Plcb1 | 1.6 | 3.1 | 1.1 | 1.9 | 1.1 | 2.4 |
| Q3V0K9 | Pls1 | 1.6 | 1.9 | 1.0 | 1.4 | 1.3 | 1.6 |
| Q8BPS4 | Gpr180 | 1.6 | 1.6 | 1.8 | 1.8 | 1.7 | 1.6 |
| P47226 | Tes | 1.6 | 4.0 | 1.2 | 3.6 | 1.3 | 3.5 |
| Q60793 | Klf4 | 1.6 | 1.9 | 1.5 | 2.0 | 1.2 | 1.5 |
| Q08509 | Eps8 | 1.5 | 3.7 | 1.4 | 3.8 | 1.5 | 3.6 |
| Q91WT8 | Rbm47 | 1.5 | 3.3 | 1.3 | 3.7 | 1.5 | 3.4 |
| P35456 | Plaur | 1.4 | 3.5 | 2.1 | 4.1 | 1.9 | 3.6 |
| P48432 | Sox2 | 1.4 | 2.8 | 1.6 | 3.1 | 2.2 | 3.5 |
| Q9ESV0 | Ddx24 | 1.4 | 2.1 | 1.6 | 2.6 | 1.4 | 2.2 |
| P47911 | Rpl6 | 1.4 | 1.6 | 1.5 | 1.8 | 1.5 | 1.8 |
| Q8K363 | Ddx18 | 1.4 | 2.0 | 1.9 | 2.7 | 1.5 | 2.3 |
| P31955 | Areg | 1.4 | 1.8 | 1.7 | 2.5 | 1.8 | 2.6 |
| Q99K82 | Smox | 1.3 | 2.1 | 1.3 | 2.5 | 1.1 | 1.7 |
| Q9QYR9 | Acot2 | 1.3 | 3.0 | 2.5 | 4.4 | 2.5 | 2.7 |
| Q9JII7 | Zfand2a | 1.3 | 2.3 | 1.2 | 2.3 | 1.1 | 2.1 |
| Q8R0F5 | Rbmx2 | 1.2 | 2.5 | 1.3 | 2.7 | 1.3 | 2.3 |
| Q8BVY0 | Rsl1d1 | 1.2 | 2.3 | 1.6 | 3.0 | 1.2 | 2.4 |
| Q8K3J5 | Znf131 | 1.2 | 1.8 | 1.3 | 2.6 | 1.3 | 2.2 |
| P12970 | Rpl7a | 1.2 | 1.9 | 1.1 | 2.0 | 1.4 | 2.3 |
| Q8CIB9 | Esco2 | 1.2 | 1.3 | 1.5 | 1.8 | 1.6 | 2.0 |
| P12032 | Timp1 | 1.2 | 2.3 | 1.5 | 3.1 | 1.4 | 2.7 |
| Q9DCA5 | Brix1 | 1.1 | 1.5 | 1.7 | 2.4 | 1.3 | 1.9 |
| Q8BHE8 | Maip1 | 1.1 | 3.0 | 1.4 | 2.6 | 1.3 | 2.9 |
| Q3UHB1 | Nt5dc3 | 1.1 | 3.7 | 1.7 | 4.6 | 1.4 | 3.6 |
| P27659 | Rpl3 | 1.1 | 1.5 | 1.4 | 2.0 | 1.3 | 1.9 |
| Q99ME9 | Gtpbp4 | 1.1 | 2.4 | 1.6 | 3.0 | 1.2 | 2.6 |
| Q9JMD1 | Sfmbt1 | 1.1 | 1.3 | 1.4 | 2.3 | 1.7 | 2.4 |
| Q9CZM2 | Rpl15 | 1.0 | 1.6 | 1.4 | 2.2 | 1.6 | 2.4 |
| P53986 | Slc16a1 | 1.0 | 3.1 | 1.4 | 3.6 | 1.5 | 3.0 |
| P62918 | Rpl8 | 1.0 | 1.6 | 1.2 | 2.0 | 1.2 | 1.9 |
| Q9CXC3 | Mgme1 | 1.0 | 2.5 | 1.8 | 4.4 | 1.6 | 3.3 |
| Q6P6L0 | Filip1l | -1.0 | 3.9 | -1.1 | 3.7 | -1.2 | 3.6 |
| Q08093 | Cnn2 | -1.0 | 3.5 | -1.2 | 3.9 | -1.2 | 4.0 |
| P56387 | Dynlt3 | -1.0 | 2.0 | -1.4 | 1.8 | -1.8 | 1.6 |
| O55222 | Ilk | -1.0 | 4.1 | -1.3 | 4.5 | -1.2 | 4.2 |
| Q01730 | Rsu1 | -1.0 | 3.8 | -1.6 | 4.3 | -1.2 | 4.2 |
| O70318 | Epb41l2 | -1.1 | 5.2 | -1.3 | 4.8 | -1.1 | 4.3 |
| Q925H1 | Trps1 | -1.1 | 2.9 | -1.4 | 3.3 | -1.2 | 3.3 |
| Q9WV92 | Epb41l3 | -1.1 | 5.5 | -1.3 | 4.6 | -1.2 | 4.0 |
| Q9Z0H4 | Celf2 | -1.1 | 2.3 | -1.4 | 4.2 | -1.3 | 3.0 |
| P35822 | Ptprk | -1.1 | 3.2 | -1.3 | 3.8 | -1.5 | 3.6 |
| P35569 | Irs1 | -1.1 | 3.7 | -1.5 | 3.7 | -1.7 | 3.6 |
| Q9CY18 | Snx7 | -1.1 | 4.5 | -1.3 | 5.0 | -1.2 | 4.5 |
| Q9R118 | Htra1 | -1.1 | 2.9 | -2.6 | 4.8 | -2.8 | 4.0 |
| F8VPU2 | Farp1 | -1.1 | 3.3 | -1.3 | 3.5 | -1.1 | 3.2 |
| P56390 | Cks2 | -1.1 | 3.5 | -1.0 | 3.1 | -1.3 | 3.4 |
| Q61553 | Fscn1 | -1.1 | 4.5 | -1.3 | 4.6 | -1.0 | 4.0 |
| P48025 | Syk | -1.2 | 3.6 | -1.5 | 4.1 | -1.1 | 4.1 |
| Q05A36 | Mex3c | -1.2 | 2.5 | -1.3 | 3.2 | -1.4 | 3.1 |
| Q91W92 | Cdc42ep1 | -1.2 | 3.0 | -1.4 | 3.6 | -1.2 | 3.1 |
| Q61234 | Snta1 | -1.2 | 4.6 | -1.2 | 3.5 | -1.2 | 4.2 |
| Q8CG19 | Ltbp1 | -1.2 | 3.8 | -1.2 | 3.7 | -1.6 | 3.8 |
| P26645 | Marcks | -1.2 | 4.0 | -1.6 | 4.0 | -1.3 | 3.9 |
| Q9CWZ7 | Napg | -1.3 | 3.1 | -1.1 | 3.3 | -1.2 | 3.3 |
| Q61062 | Dvl3 | -1.3 | 2.0 | -1.2 | 1.3 | -2.5 | 1.8 |
| Q9ESL4 | Map3k20 | -1.3 | 4.0 | -1.5 | 4.7 | -1.4 | 3.9 |
| P62965 | Crabp1 | -1.3 | 3.7 | -1.8 | 3.7 | -1.5 | 3.4 |
| Q8CC35 | Synpo | -1.3 | 3.6 | -1.9 | 3.7 | -1.7 | 3.1 |
| Q6PCZ4 | Magee1 | -1.3 | 2.1 | -1.1 | 1.3 | -2.0 | 2.0 |
| Q922P8 | Tmem132a | -1.3 | 2.8 | -1.7 | 3.5 | -1.7 | 2.6 |
| O88942 | Nfatc1 | -1.4 | 2.0 | -1.3 | 3.3 | -1.3 | 2.8 |
| Q9R1B9 | Slit2 | -1.4 | 4.0 | -1.2 | 3.6 | -1.4 | 4.2 |
| Q8BQ89 | Lix1l | -1.4 | 1.4 | -2.2 | 3.7 | -2.2 | 1.4 |
| Q63918 | Cavin2 | -1.4 | 3.4 | -1.0 | 3.5 | -1.3 | 2.5 |
| P61588 | Rnd3 | -1.4 | 3.6 | -1.2 | 2.8 | -1.7 | 4.0 |
| P0DOV2 | Ifi204 | -1.4 | 2.6 | -3.0 | 2.0 | -2.0 | 2.3 |
| P97315 | Csrp1 | -1.4 | 4.5 | -1.6 | 4.9 | -1.6 | 3.4 |
| Q91ZJ5 | Ugp2 | -1.5 | 4.3 | -1.8 | 4.8 | -1.7 | 4.5 |
| Q9JKS5 | Habp4 | -1.5 | 3.1 | -2.5 | 2.0 | -2.5 | 3.6 |
| Q05816 | Fabp5 | -1.5 | 3.9 | -1.9 | 3.6 | -1.8 | 3.7 |
| Q80YS6 | **Afap1** | -1.5 | 2.8 | -1.9 | 3.8 | -1.7 | 3.3 |
| Q8K1N2 | Phldb2 | -1.5 | 4.4 | -2.1 | 4.0 | -2.1 | 4.0 |
| O70456 | Sfn | -1.5 | 2.1 | -2.3 | 1.5 | -2.4 | 1.5 |
| Q9CQW9 | Ifitm3 | -1.5 | 3.2 | -1.2 | 2.9 | -1.7 | 2.9 |
| Q61823 | **Pdcd4** | -1.6 | 3.5 | -1.6 | 3.1 | -1.9 | 3.7 |
| P48036 | Anxa5 | -1.6 | 1.7 | 1.0 | 3.3 | 1.1 | 4.1 |
| Q8R3B1 | Plcd1 | -1.6 | 2.5 | -1.4 | 3.5 | -1.3 | 3.0 |
| P98203 | Arvcf | -1.6 | 2.4 | -1.8 | 3.1 | -1.3 | 2.7 |
| P22777 | Serpine1 | -1.6 | 3.2 | -1.5 | 2.7 | -2.0 | 3.6 |
| P29533 | Vcam1 | -1.7 | 3.1 | -1.5 | 3.1 | -1.5 | 3.1 |
| Q61572 | Foxc1 | -1.7 | 3.0 | -1.4 | 4.0 | -1.2 | 3.1 |
| Q08879 | Fbln1 | -1.7 | 3.8 | -1.7 | 2.6 | -1.7 | 2.5 |
| P51655 | Gpc4 | -1.7 | 4.8 | -1.0 | 3.6 | -1.3 | 4.7 |
| Q8BLX7 | Col16a1 | -1.7 | 2.2 | -3.4 | 3.8 | -3.6 | 2.6 |
| P20357 | Map2 | -1.7 | 2.9 | -2.5 | 4.0 | -2.2 | 4.0 |
| Q61292 | Lamb2 | -1.7 | 3.3 | -1.5 | 3.4 | -1.4 | 3.2 |
| P54254 | Atxn1 | -1.7 | 2.7 | -2.4 | 3.4 | -1.9 | 1.5 |
| Q7TN75 | Peg10 | -1.7 | 1.6 | -2.0 | 3.3 | -2.2 | 1.8 |
| Q9CWS0 | Ddah1 | -1.8 | 4.6 | -2.4 | 4.8 | -2.2 | 4.2 |
| Q61398 | Pcolce | -1.8 | 3.6 | -1.5 | 3.4 | -1.4 | 2.8 |
| Q4KML4 | Abracl | -1.8 | 4.2 | -1.4 | 3.6 | -1.8 | 4.3 |
| P15066 | Jund | -1.8 | 1.9 | -1.9 | 1.6 | -1.7 | 1.4 |
| Q99K41 | Emilin1 | -1.8 | 3.7 | -2.1 | 4.4 | -2.1 | 3.8 |
| Q8BU85 | Msrb3 | -1.8 | 3.7 | -1.6 | 3.0 | -1.8 | 4.3 |
| Q9CWF2 | Tubb2b | -1.9 | 3.5 | -1.5 | 3.5 | -1.3 | 4.1 |
| Q8K2A1 | Gulp1 | -1.9 | 4.0 | -2.0 | 4.7 | -1.9 | 4.4 |
| Q9WVJ9 | Efemp2 | -1.9 | 4.0 | -1.5 | 3.9 | -1.5 | 3.4 |
| Q8BH64 | Ehd2 | -1.9 | 4.3 | -2.3 | 4.6 | -2.0 | 3.9 |
| Q8K262 | Plac9 | -1.9 | 1.3 | -1.5 | 1.5 | -1.8 | 1.4 |
| Q8VHY0 | Cspg4 | -1.9 | 3.7 | -1.8 | 4.2 | -1.7 | 4.0 |
| Q05793 | Hspg2 | -2.0 | 4.7 | -1.8 | 3.8 | -1.6 | 3.9 |
| Q9JJE7 | Fads3 | -2.0 | 2.9 | -2.0 | 2.8 | -1.8 | 3.3 |
| Q62000 | Ogn | -2.0 | 1.7 | -1.2 | 2.3 | -2.2 | 3.5 |
| Q9CYL5 | Glipr2 | -2.1 | 4.7 | -2.1 | 3.7 | -1.7 | 3.9 |
| O35309 | Nmi | -2.1 | 2.7 | -2.0 | 2.6 | -1.2 | 2.9 |
| Q9D032 | Ssbp3 | -2.1 | 3.8 | -1.8 | 4.1 | -2.0 | 4.1 |
| Q61554 | Fbn1 | -2.1 | 3.7 | -1.7 | 3.6 | -2.1 | 3.5 |
| O89026 | Robo1 | -2.1 | 2.4 | -2.1 | 3.1 | -3.3 | 3.6 |
| Q9ESD7 | Dysf | -2.1 | 2.2 | -1.9 | 2.5 | -2.1 | 2.8 |
| P58771 | Tpm1 | -2.1 | 4.5 | -1.8 | 5.2 | -2.2 | 4.3 |
| Q5BKP2 | Usp13 | -2.1 | 1.7 | -1.9 | 1.7 | -2.7 | 2.1 |
| Q9ET54 | **Palld** | -2.1 | 4.5 | -2.1 | 5.0 | -1.9 | 4.5 |
| Q8VDF3 | **Dapk2** | -2.2 | 2.2 | -2.6 | 1.9 | -3.4 | 2.6 |
| P07214 | Sparc | -2.2 | 3.8 | -1.6 | 3.7 | -1.7 | 3.5 |
| Q01721 | Gas1 | -2.2 | 2.9 | -2.5 | 2.8 | -2.2 | 2.0 |
| Q8JZX9 | Cdc42ep2 | -2.2 | 1.8 | -2.1 | 2.6 | -2.0 | 1.9 |
| O54775 | Ccn4 | -2.2 | 2.0 | -2.8 | 2.8 | -2.9 | 2.6 |
| Q99LJ6 | Gpx7 | -2.2 | 3.2 | -1.2 | 2.8 | -1.4 | 2.6 |
| Q60760 | Grb10 | -2.3 | 3.9 | -2.3 | 4.4 | -2.6 | 3.4 |
| Q9Z1R3 | Apom | -2.3 | 1.5 | -1.5 | 1.6 | -2.5 | 3.3 |
| Q8R1S9 | Slc38a4 | -2.3 | 3.1 | -3.9 | 2.4 | -3.0 | 2.3 |
| Q9CQE5 | Rgs10 | -2.3 | 2.0 | -2.0 | 1.7 | -3.1 | 3.3 |
| Q60648 | Gm2a | -2.3 | 2.5 | -1.6 | 2.7 | -1.8 | 2.8 |
| P28231 | Gjb3 | -2.3 | 1.9 | -1.5 | 2.9 | -2.5 | 3.4 |
| O08573 | Lgals9 | -2.3 | 2.5 | -1.9 | 3.5 | -2.4 | 2.4 |
| Q6NSU3 | Glt8d1 | -2.4 | 1.9 | -1.9 | 1.6 | -1.1 | 1.3 |
| Q9JJN6 | Ctnnbip1 | -2.4 | 2.0 | -2.4 | 1.5 | -2.4 | 1.3 |
| Q01063 | Pde4d | -2.4 | 2.9 | -2.3 | 3.0 | -1.8 | 1.8 |
| Q8BH97 | Rcn3 | -2.4 | 3.4 | -1.6 | 3.3 | -1.9 | 3.2 |
| Q8BWL5 | Rbms3 | -2.4 | 1.6 | -2.9 | 2.1 | -3.2 | 1.9 |
| P48758 | Cbr1 | -2.4 | 3.3 | -2.3 | 4.4 | -2.1 | 3.6 |
| O88207 | Col5a1 | -2.4 | 2.7 | -2.2 | 3.2 | -2.3 | 3.0 |
| O35955 | Psmb10 | -2.5 | 2.7 | -2.6 | 3.7 | -3.3 | 2.8 |
| Q9WUU7 | Ctsz | -2.5 | 2.0 | -1.3 | 2.0 | -1.8 | 2.6 |
| Q8CG71 | P3h2 | -2.5 | 2.1 | -2.8 | 3.5 | -2.6 | 2.1 |
| Q6Q477 | Atp2b4 | -2.5 | 2.3 | -2.2 | 3.3 | -1.8 | 1.7 |
| P16460 | Ass1 | -2.5 | 1.4 | -1.4 | 3.6 | -1.8 | 2.2 |
| Q62407 | Speg | -2.5 | 1.8 | -4.3 | 3.3 | -4.1 | 2.8 |
| Q9Z191 | Eya4 | -2.5 | 2.5 | -2.5 | 2.2 | -2.5 | 2.6 |
| P37804 | Tagln | -2.6 | 2.3 | -1.8 | 3.6 | -3.2 | 2.2 |
| P58022 | Loxl2 | -2.6 | 4.6 | -2.0 | 2.6 | -1.8 | 2.4 |
| Q61245 | Col11a1 | -2.7 | 2.2 | -3.2 | 1.8 | -3.2 | 3.4 |
| Q570Y9 | Deptor | -2.7 | 3.3 | -3.4 | 2.7 | -4.4 | 3.9 |
| Q62219 | Tgfb1i1 | -2.8 | 1.6 | -2.0 | 1.4 | -2.6 | 2.9 |
| P40224 | Cxcl12 | -2.9 | 2.7 | -2.9 | 2.9 | -3.1 | 3.0 |
| Q9JLM8 | Dclk1 | -2.9 | 1.5 | -1.4 | 2.7 | -2.1 | 2.9 |
| Q9CQ19 | Myl9 | -3.0 | 3.0 | -3.0 | 4.8 | -3.2 | 4.1 |
| Q9DC11 | Plxdc2 | -3.0 | 2.8 | -3.7 | 1.5 | -3.2 | 2.7 |
| Q04857 | Col6a1 | -3.0 | 3.2 | -1.8 | 4.1 | -2.5 | 3.5 |
| Q3U962 | Col5a2 | -3.0 | 4.0 | -2.5 | 4.1 | -3.0 | 3.6 |
| O08736 | **Casp12** | -3.1 | 1.6 | -4.0 | 3.3 | -4.6 | 3.2 |
| Q61581 | Igfbp7 | -3.3 | 1.9 | -2.2 | 3.6 | -3.1 | 2.5 |
| P11276 | Fn1 | -3.3 | 5.2 | -2.8 | 4.0 | -2.2 | 3.7 |
| Q8BTG3 | Tcp11l1 | -3.3 | 1.8 | -3.7 | 1.6 | -2.5 | 2.4 |
| Q02788 | Col6a2 | -3.5 | 3.6 | -3.6 | 2.1 | -3.7 | 2.7 |
| Q61391 | Mme | -3.6 | 1.6 | -1.3 | 2.9 | -1.2 | 2.4 |
| Q8K157 | Galm | -3.7 | 2.2 | -3.9 | 2.0 | -3.1 | 3.4 |
| P37889 | Fbln2 | -3.7 | 5.2 | -3.5 | 5.2 | -3.9 | 4.6 |
| P11087 | Col1a1 | -3.7 | 4.2 | -3.8 | 4.4 | -3.9 | 4.5 |
| Q9JHT5 | Ammecr1 | -3.7 | 4.0 | -4.3 | 2.3 | -5.1 | 2.9 |
| Q9JHI5 | Ivd | -3.7 | 3.8 | -4.3 | 3.1 | -3.7 | 1.6 |
| P97873 | Loxl1 | -3.9 | 4.1 | -2.7 | 4.4 | -2.7 | 3.3 |
| Q9Z2V5 | Hdac6 | -3.9 | 3.7 | -5.1 | 2.7 | -3.8 | 3.6 |
| Q8K4G5 | **Ablim1** | -3.9 | 2.9 | -2.9 | 2.8 | -2.9 | 2.7 |
| Q91XV3 | Basp1 | -4.0 | 4.6 | -4.2 | 5.0 | -3.8 | 4.8 |
| Q8VEB1 | Grk5 | -4.1 | 2.3 | -3.6 | 2.7 | -3.9 | 3.2 |
| Q5RL51 | Gstcd | -4.2 | 1.7 | -2.5 | 3.1 | -3.1 | 2.6 |
| Q91ZE0 | Tmlhe | -4.3 | 3.1 | -3.7 | 2.5 | -4.3 | 3.3 |
| Q8K007 | Sulf1 | -4.3 | 1.9 | -3.1 | 4.8 | -3.3 | 4.1 |
| P28301 | Lox | -4.5 | 3.0 | -4.2 | 3.4 | -3.6 | 3.5 |
| Q924W7 | Dennd2b | -4.6 | 2.2 | -2.7 | 1.6 | -3.2 | 1.5 |
| P97447 | Fhl1 | -4.8 | 5.8 | -4.8 | 5.5 | -4.5 | 4.6 |
| P01831 | Thy1 | -4.9 | 3.8 | -4.4 | 5.2 | -5.4 | 3.0 |
| Q64133 | Maoa | -5.1 | 4.4 | -5.5 | 4.0 | -5.3 | 3.9 |
| Q8BR92 | Pakap | -5.1 | 3.1 | -5.1 | 3.4 | -4.0 | 2.1 |
| P70180 | Npr3 | -5.3 | 2.0 | -4.4 | 3.7 | -4.9 | 4.2 |

**a** Cellular protein signature of MPXV MOI 1 infected MEF lysates at 21 hours post-infection. Protein ID, protein name, gene name, log2-fold change (FC) (MPXV infected MEF versus MEF Mock) of the levels of each protein, and statistical significance (−log P value) of each Clade II strain are listed. Genes mentioned in the text are highlighted in bold.
